# Nitrogen Loading Enhances Stress Impact of Drought on a Semi-natural Temperate Grassland

**DOI:** 10.3389/fpls.2019.01051

**Published:** 2019-08-30

**Authors:** Angelika Kübert, Miriam Götz, Emma Kuester, Arndt Piayda, Christiane Werner, Youri Rothfuss, Maren Dubbert

**Affiliations:** ^1^Ecosystem Physiology, Freiburg University, Freiburg, Germany; ^2^Thünen Institute of Climate-Smart Agriculture, Braunschweig, Germany; ^3^Institute of Bio- and Geosciences, Agrosphere Institute (IBG-3), Forschungszentrum Jülich, Jülich, Germany

**Keywords:** nitrogen loading, extreme drought, synergistic effects, stress response, grassland, ecosystem fluxes, partitioning, water use efficiency

## Abstract

Two important threats to the sustainable functioning of seminatural grasslands in temperate zones are (1) nutrient loading due to agricultural fertilization and pollution, and (2) the increase of extreme drought events due to climate change. These threats may cause substantial shifts in species diversity and abundance and considerably affect the carbon and water balance of ecosystems. The synergistic effects between those two threats, however, can be complex and are poorly understood. Here, we experimentally investigated the effects of nitrogen addition and extreme drought (separately and in combination) on a seminatural temperate grassland, located in Freiburg (South Germany). To study the grassland response, we combined eddy-covariance techniques with open gas exchange systems. Open gas exchange chambers were connected to an infrared gas analyzer and water isotope spectrometer, which allowed the partitioning of net ecosystem exchange and evapotranspiration. Vegetation parameters were described by species richness, species abundance, and leaf area index. Our results suggest that grassland communities, strongly weakened in their stress response by nitrogen loading, can substantially lose their carbon sink function during drought. While nitrogen addition caused a significant loss in forb species (−25%), precipitation reduction promoted a strong dominance of grass species at season start. Consequently, the grass-dominated and species-poor community suffered from a strong above-ground dieback during the dry summer months, likely caused by lower water use efficiency and weaker drought adaptations of the species community. Over the growing season (April-September), the carbon sequestration of the studied grassland was reduced by more than 60% as a consequence of nitrogen addition. Nitrogen addition in combination with precipitation reduction decreased carbon sequestration by 73%. Eutrophication can severely threaten the resilient functioning of grasslands, in particular when drought periods will increase as predicted by future climate scenarios. Our findings emphasize the importance of preserving high diversity of grasslands to strengthen their resistance against extreme events such as droughts.

## Introduction

Many ecosystems are experiencing major threats for their sustainable functioning such as climate change, land use change, and pollution (Grimm et al., 2008; IPBES, 2018). Particularly, the degradation and loss of grassland ecosystems pose a special threat. Natural and seminatural grasslands provide important ecosystem services such as biodiversity preservation, carbon sequestration, and forage production (White et al., 2000; Millennium Ecosystem Assessment, 2005; Stoate et al., 2009; Schnyder et al., 2010; Maestre et al., 2012). Worldwide, grasslands cover about 40% of the Earth’s terrestrial surface (excluding Greenland and Antarctica, White et al., 2000) and are among the dominant agricultural land-use types in Europe with 30% of the agriculturally used area (Stoate et al., 2009). While at a larger scale temperate European ecosystems are relatively poor in species diversity, seminatural temperate grasslands offer a hotspot of biodiversity on a small scale (Habel et al., 2013; Dengler et al., 2014). Since the 1950s, however, seminatural temperate grasslands in Europe have been strongly declining in extent and biodiversity (Dengler et al., 2014; IPBES, 2018; Török and Dengler, 2018). 

Nitrogen loading and drought stress are two important drivers in grassland ecosystems that can substantially influence the ecosystem functioning across different scales (from the single plant to the community level) (e.g., Wedin and Tilman, 1996; Jacquemyn et al., 2003; Ciais et al., 2005; Jentsch and Beierkuhnlein, 2008; Grant et al., 2014). Human activities, such as fertilizer runoff and environmental pollution, have globally a severe impact on the nitrogen cycle of ecosystems (Galloway, 1995; Steffen et al., 2015; IPBES, 2018). Especially the effect of pollution, as a major driver for the loss of biodiversity and ecosystem services, has strongly increased in the last 20 years (IPBES, 2018). Nitrogen loading was found to significantly decrease biodiversity in (nitrogen-limited) grasslands through altered competition between species (Wedin and Tilman, 1996; Stevens, 2004; Stevens et al., 2006; Harpole and Tilman, 2007; Hautier et al., 2009).When, due to nitrogen loading, nitrogen supply is not limited anymore in an ecosystem, another resource may become the new limiting and key resource to compete for (Tilman 1982). The competition for the resource light, for instance, was found as the main mechanism for diversity loss in fertilized experimental grassland communities (Hautier, 2009). Nitrogen loading may favor species with specific functional traits, such as tall stature to compete for light, and cause the competitive exclusion and loss of other species (Tilman et al., 1997). A higher species diversity, on the other hand, was found to be beneficial for the stable functioning of grasslands (Tilman and Downing, 1994). A lower diversity, due to nitrogen loading, could increase the grassland community’s sensitivity to extreme events such as drought. 

Extended drought periods are expected to increase in the future. Global climate change projections predict an increase in droughts for Central Europe, increasing both their intensity and duration (Kovats et al., 2014; Spinoni et al., 2018). Shallow rooted grasslands suffer particularly from the consequences of drought due to the direct dependency of ecosystem productivity on precipitation (Leitinger et al., 2015). Summer drought was, for instance, found to decrease above-ground plant productivity. This negative impact was, however, independent from species diversity (Kahmen et al., 2005b). Further, it has been shown that a strong change in precipitation patterns (not amount) during the growing season, resulting in an extreme drought followed by heavy rainfalls, strongly reduced grassland forage yield. This negative effect observed in this study was likely related to changes in the community composition (Grant et al., 2014). On the other hand, grasslands were found to have a stronger response to years with high rainfall, that is, a stronger increase in the aboveground net primary production in wet years than decrease in it in dry years (Knapp and Smith, 2001). This asymmetric response might indicate a relative low sensitivity of grassland ecosystems to drought events. Nevertheless, the high variation in aboveground net primary production that grasslands experience might make them more susceptible to drought events occurring at sensitive growth stages, in particular following high rainfall periods. 

In the past, several experimental approaches have been conducted studying the impact of simulated extended drought periods, altered precipitation regimes, or fertilization with different macronutrients and micronutrients on (temperate) grassland communities. Such manipulation experiments have been established as “natural laboratories,” enabling a process-based understanding of vegetation drought and nutrient responses (De Boeck et al., 2015). However, only few studies dealt with the combined effect of (simulated) drought and nitrogen loading on temperate grasslands (Grant et al., 2014). The interactions between these two factors, however, can be complex and are largely unknown (Lü et al., 2014). In addition, the main focus of these studies typically was set on the response in productivity, that is, biomass production, structure, and biodiversity of plant communities (Wedin and Tilman, 1996; Grime et al., 2000; Pfisterer and Schmid, 2002; Kahmen et al., 2005a; Jentsch and Beierkuhnlein, 2008; Gilgen and Buchmann, 2009; Socher et al., 2013), but also carbon dioxide or methane emissions (Fay et al., 2004; Jentsch et al., 2011). Changes in the species composition, however, should also affect ecosystem carbon and water fluxes. Ecosystem carbon and water fluxes integrate the fluxes of all above- and below-ground processes; that is, they include all species-related gas exchange processes such as transpiration, respiration, and photosynthetic rates, but also evaporative loss from species biomass surface (Potts et al., 2006; Harpole et al., 2007). Species, however, can substantially differ in their morphology, phenology, and physiology, influencing their response to a changing environment. This way, changes in the species composition translate to the ecosystem level and affect water and carbon fluxes of the ecosystem. 

A high uncertainty still exists regarding the ecohydrological responses, that is, the responses of the close link between water and carbon fluxes of grassland communities to droughts (Rodriguez-Iturbe, 2000). Water relations of the vegetation and ecosystem water cycling, however, are the primary factors that are influenced by droughts. In particular, the interplay between changes in species composition and abundance, the interspecific competition balance, and plant-to-community scale ecophysiological adaptations to a changing environment remain unclear. Different species have different strategies and adaptations to drought stress, but also different life cycle strategies (e.g., Craine et al., 2012). Net primary production of many terrestrial ecosystems is limited by nitrogen availability (LeBauer and Treseder, 2008). Moreover, an increase of nitrogen availability (Singh et al., 1975; Taylor et al., 1991) but also prolonged drought stress may lead to increased water use efficiencies of plant species, directly influencing gross ecosystem carbon and water budgets. Increasing their water use efficiency is one mechanism of plant species to cope with water stress in order to maximize energy production and reduce water costs (Farquhar and Sharkey, 1982). Ponce-Campos et al. (2013) found that across all biomes, including grasslands, ecosystem water use efficiencies significantly increased with drought. In temperate grassland specifically, increased ecosystem water use efficiencies were observed in response to summer drought (Wever et al., 2002), but also no changes in response to spring drought (Wolf et al., 2013). However, under extreme prolonged drought, temperate grasslands might be forced to increase their water use efficiency due to long-term limited water availability. 

In order to better understand the close link between water availability, plant productivity, and species composition, we studied the combined effect of nutrient addition and water limitation on the carbon and water fluxes of a temperate grassland ecosystem. We particularly focused on the seasonal dynamics of carbon and water fluxes in relation to changing environmental conditions and vegetation structures. The studied grassland system is part of the Nutrient Network (www.nutnet.org) and the International Drought Net (www.drought-net.org). Following the networks’ protocols, nitrogen fertilization was applied in spring and year-round rain-out shelters set up in order to simulate conditions of nitrogen loading and a one in 100-year drought. First analysis within the International Drought Net showed that precipitation reduction (i.e., drought) significantly reduced minimum soil water content and above-ground biomass (mainly in forbs) at the experimental site in Freiburg (Henry et al., 2018). 

In order to quantify the carbon and water fluxes in our system, we combined eddy-covariance techniques with gas exchange systems, connected with an infrared gas analyzer for carbon fluxes and a cavity ring down spectrometer for isotopic analyses. Using stable isotopes in combination with gas exchange chambers and eddy-covariance techniques can provide long-term and high-resolution ecosystem carbon and water fluxes and allow to partition evapotranspiration (Hornberger, 1999; Werner et al., 2012; Dubbert et al., 2013, Dubbert et al., 2014; Rothfuss and Javaux, 2017). Vegetation parameters were described by species richness, species abundance and leaf area index (LAI). We hypothesized that (I) precipitation reduction will strongly reduce plant production and consequently the capacity of sequestrating carbon by the studied grassland system due to water limitations; (II) nitrogen addition will promote plant production since nitrogen is a limiting resource for growth in the grassland system; (III) in combination, nitrogen addition will consequently counteract the negative impact of drought stress on the carbon sink functioning by increasing plant production. 

## Materials and Methods

### Study Site

Measurements took place in a perennial seminatural temperate grassland with a shallow root system (> 95% of bulk root biomass in 0-20 cm, root profile of untreated grassland area: 0-45 cm, n = 4) and no access to ground water (ground water level at approximately 10-m depth). The study site is located in the direct neighborhood of the technical faculty campus of Freiburg University (48°1′22″N; 7°49′57″O, elevation: 238 m a.s.l.). The studied system is a seminatural and ruderal grassland system, dominated by grass species. The species community consists of 59 species; characteristic species are *Agrostis capillaris* L., *Carex hirta* L., *Centaurea jacea* L., *Dactylis glomerata* L., *Festuca pratensis* HUDS, and *Festuca rubra* L. (all grass species, but *C. jacea*, which is a dicot species). The soil can be characterized as an anthrosol; it contains lessivated brown earth as a top layer, whereas lower layers are built up by crushed stone (LGRB, 2017). The studied site was subject to denudation and landfill, but the system was rebuilt and has been allowed to recover for 4 years before measurements started. Until 2015, it was extensively grazed by sheep, once a year at the end of June/beginning of July. The long-term (1987-2016) average of annual temperature and rainfall are 11.3 °C and 901.8 mm, with 177.9 mean days of rainfall per year (Deutscher Wetterdienst DWD, 2018; weather station in approximately 700 m distance to field site).

### Experimental Design

The experimental field site combined the design of the two international monitoring networks Drought Net and Nutrient Network. The International Drought Net aims at assessing the effects of a (simulated) one in a 100-year drought on different ecosystems (short- and tall-stature vegetation). The Nutrient Network is focused on the effects of different nutrient loading on grassland communities worldwide. The combined design of our field site, the Drought Nut Net design, imposes four distinct treatments on the grassland field site: (I) natural nitrogen availability (control) and ambient precipitation (control) (CC), (II) natural nitrogen availability and precipitation reduction (CD), (III) nitrogen addition with ambient precipitation (NC), and (IV) nitrogen addition with precipitation reduction (ND). Each treatment (control control [CC], control drought [CD], nitrogen control [NC], nitrogen drought [ND]) is replicated four times, that is, four plots for four treatments, in total 16 experimental plots. Plots were installed (10 × 5 m) in autumn 2015 for premeasurements, followed by the experiment start (setup of rain-out shelters and nitrogen addition) in spring 2016. Premeasurements were carried out in order to guarantee for comparability between plots and treatments, including species composition and biomass (above and belowground), soil C and N content as well as C/N ratios, soil properties, and water content profiles. No statistical differences between treatments could be found (T. Gebauer, personal communication). Drought stress is experimentally simulated by year-round rain-out shelters, installed above half of each plot (4 × 3 m), with a 1-m gap in between subplots as buffer. Transparent shelter material was placed in stripes (5 times 32 cm) above the canopy (in 120- to 160-cm height) in order to minimize impacts on micrometeorological conditions [mean reduction in mean photosynthetic photon flux density (PPFD) by roofs: –71 µmol m^−2^s^−1^ (–19%), mean reduction in air temperature: –0.4 °C (–2%), mean increase in air relative humidity: +1% (+1%), in 2016]. The precipitation reduction simulates a 100-year drought, that is, an extreme drought defined for this study site as a reduction in annual precipitation by approximately 36% (Knapp et al. 2017, International Drought Experiment protocol). The area covered by rain-out shelters was set higher (i.e., to 40%) in order to account for lateral precipitation input. A test of the shelters showed an effective reduction in precipitation by 36.7% ± 8.2% (tested events: heavy rain, long steady rain, rain shower). Precipitation is collected and directed away from the plots by pipes. Nitrogen fertilization started in spring 2016; a granular MultiCote 40% urea fertilizer (N_2_H_4_CO; Haifa Chemicals Ltd., Haifa, Israel) with a 4-month release was applied. Nitrogen plots are treated with the fertilizer once a year in spring adding 10 g N m^−2^ y^−1^ by elementals mass to the plots (specification in Nut Net protocol). This rate is relatively high but comparable to previous studies (Nut Net protocol).

### Environmental Data

Soil volumetric water content (VWC, 10HS, Decagon, WA, USA) and soil temperature (T106, T108, Campbell Scientific, Logan, UT, USA) in 1-, 5-, 20-, and 40-cm depth were measured (4 treatments, 4 replicates, 4 depths, in total 64) and stored as 30-min averages in data loggers (CR1000; Campbell Scientific, Logan, UT, USA). Precipitation (RG3-M HOBO rain gauge; Onset, Bourne, MA, USA), air temperature, air relative humidity (S-THB-M008, Onset), and PPFD (S-LIA-M003; Onset) were measured above canopy (1-m height) every 30 minutes and stored in HOBO data loggers (HOBO H21-002 & U30, Onset). Vapor-pressure deficit was calculated by using the August-Roche-Magnus equation for the saturated vapor pressure (Alduchov and Eskridge, 1996). Long-term climate data were available from a weather station (Airport Freiburg) in approximately 700-m distance to field site, run by the German weather forecast service DWD. Data gaps in precipitation measurements were filled by data from this station.

### Vegetation Parameters

Vegetation parameters of experimental plots were assessed from April to September covering the growing season of the grassland ecosystem. Data on vegetation parameters, that is, species richness, species abundance, and LAI, were collected once per month, in May and July twice, which means in total 8 measurement campaigns. For vegetation measurements, a random subplot (2 × 1.5 m) was designated of each of the 16 plots (n = 4 per treatment). This subplot was used for measurements for the whole growing season. The species present on this subplot were assessed and their absolute canopy cover of the subplot estimated. We pooled all sweet-grass species as *Poaceae*. Species richness was defined as species present per subplot. Relative species abundance/cover was estimated as the percentage of species on total canopy cover on each subplot. For further analysis, species were pooled into the functional groups grasses and forbs and the absolute/relative cover of functional groups derived. The LAI was measured with the LAI-2000 Plant Canopy Analyzer (Li-Cor Biosciences, Lincoln, NE, USA) under constant atmospheric conditions. One atmospheric capture and three below canopy captures were taken three times on each subplot. If the standard error was >0.7, the measurement was repeated. The LAI was calibrated against the leaf area of biomass harvests. 

### Carbon and Water Fluxes 

#### Chamber-Based Flux and Isotopic Measurements

Chamber-based flux and isotopic measurements were conducted on the experimental plots from April to September covering the grassland growing season. Fluxes and isotopic signature of evapotranspiration were measured by coupling a cavity ring-down spectrometer (*CRDS*, L2120-i; Picarro Inc., Santa Clara, CA, USA) and infrared gas analyzer (Li820; Li-Cor Biosciences) to custom-built open gas exchange chambers [design after Pape et al. (2009), successfully tested by Dubbert et al. (2013)]. The chambers are two cylindrical, transparent acrylic chambers (60 L, base area 962 cm^2^) coated with FEP foil (4PTFE, Stuhr, Germany) with an inlet (atmospheric air stream) and outlet port (sampling air). A fan inside the inlet port regulates the flow through the chambers between 0 and 40 L min^−1^ and fully mixes the air inside the chamber. Both gas exchange chambers were equipped with sensors for air temperature, air relative humidity, PPFD, and flow rate, storing every minute in a data logger (HOBO U12-013, Onset; LI-190R, AWM720P1; Honeywell, Morris Plains, NJ, USA). For measurements, chambers were placed on metallic rings to avoid damage of vegetation and to achieve air tightness. The chambers stayed on the rings until stabilization in gas exchange was reached (< 15 min). The metallic rings (Ø 35 cm) were installed on a random subplot (different from the one for the vegetation parameters) in spring 2016. Due to this labor-intensive type of measurements, only 3 plots per treatment could be included (n = 3 per treatment, in total 12 plots). A complete set of daytime fluxes of all treatments was collected within 2 consecutive days (2 days similar in environmental conditions) by alternating the two chambers between the 12 plots (6 plots per each day). Parallel to chamber measurements, soil and plant material was collected for isotopic analysis, which was needed for the evapotranspiration partitioning approach. Isotopic signatures of evapotranspiration were determined by mass balance [see Appendix A “Methods: Isotopic Signatures of Evapotranspiration, Leaf and Soil Samples” and Appendix B “Isotope Theory”; Supplementary Figure 1 (**Supplementary Figure 1**) in **Supplementary Material**]. Chamber measurements took place every 3 to 4 weeks, parallel to vegetation-related measurements. They were intensified in July with weekly measurements; that is, in total 10 chamber measurements campaigns took place during the growing season. 

Before measurements, the *CRDS* was calibrated against three laboratory standards (light, middle, heavy) by liquid water injection into the vaporizer of the analyzer. Laboratory standards were calibrated against V-SMOW, SLAP, and GISP (IAEA, Vienna). During field measurements, the *CRDS* was calibrated by using a custom-built soil water vapor standard, a laboratory container filled with quartz sand (0.3-0.8 mm) after the method of Rothfuss et al., 2013. This method is based on gas-permeable polypropylene tubes that equilibrate with the soil water. The soil water vapor standard was sampled every 8 hours for 14 min. The infrared gas analyzer was calibrated against a laboratory calibrated gas exchange system GFS 3000 (Walz, Effeltrich, Germany). Water (carbon) fluxes were calculated as differences in H_2_O (CO_2_) mixing ratios, that is, the deviation between inlet and outlet air sample. Ecosystem respiration (R_eco_) was measured by darkening the chambers for at least 5 minutes. Evapotranspiration (ET), net ecosystem exchange (NEE), gross primary production (GPP), and ecosystem respiration (R_eco_) were calculated as in von Caemmerer and Farquhar (1981). In this study, NEE < 0 indicates carbon sequestration from the atmosphere (i.e., carbon sink) by the ecosystem. 

#### Partitioning of Carbon and Water Fluxes 

Evapotranspiration, NEE, and *R*_eco_ of chamber-based measurements were calculated after von Caemmerer and Farquhar (1981). The GPP is derived by subtracting *R*_eco_ from NEE. The fraction of transpiration *ft* = T/ET was calculated with measured ET and ∂^18^O_ET_ and modeled ∂^18^O*_E_* and ∂^18^O*_T_* (for modeling theory see Appendix A in **Supplementary Material**: Isotope Theory; Moreira et al., 1997; Yakir and Sternberg, 2000): 

(1) $$ft=\frac{T}{ET}=\frac{\delta_{ET}-\delta_{E}}{\delta_{T}-\delta_{E}}$$

Averages of measured fluxes were derived by locally estimated scatterplot smoothing (loess); standard deviations were calculated as 68% confidence interval (see Appendix A in **Supplementary Material** for more details). Important assumptions for the calculations of daily sums of fluxes were that T and GPP are 0 at the time of sunrise and sunset and during the night. A 1-hour mean value for E and R_eco_ at the start and end of the measurements was calculated in order to extrapolate for the whole night. During night, NEE equals R_eco_, and ET equals E. 

#### Eddy-Covariance–Based Flux Measurements

H_2_O and CO_2_ fluxes of the treatment CC (ambient) were continuously measured by an eddy-covariance system. The eddy-covariance system was set up on a 3-m-high tower aside from the sheltered and fertilized area (distance to the experimental plots for chamber and vegetation measurements: 41-85 m). The footprint of the eddy-covariance system only included untreated grassland area (on average 1600 m^2^), which resampled the treatment CC of the experimental plots. The eddy-covariance system consisted of an open-path infrared gas analyzer (Li-7500A; Li-Cor Biosciences) and a 3D sonic anemometer (R3, Gill Instruments Ltd., Lymington, UK). Collected data were continuously recorded by EddyMeas (Meteotools, Jena, Germany) (Kolle and Rebmann, 2007). Relative air humidity, air temperature (HMP15; Vaisala, Vantaa, Finland), and solar radiation (CNR1 net radiation meter, PQS1 PAR sensors; Kipp & Zonen, Delft, the Netherlands) were collected every 30 min. Atmospheric pressure was acquired from a nearby weather station (DWD, Airport Freiburg, approximately 700 m NE of the field site). Eddy data processing was done using EddySoft (Meteotools) (Kolle and Rebmann, 2010) and Python2.7 scripts (Python Software Foundation, Wilmington, DE, USA). A detailed description of processing steps can be found in Piayda et al. (2014). Ahead of flux gap filling, footprint analysis results of EddySoft were used to exclude any flux impacted by the treatment area.

#### Seasonal Budget of Carbon and Water Fluxes

Continuous carbon and water fluxes during the growing season (April to September) were determined by matching continuous eddy-covariance–based flux data to chamber-based flux data. First, correlations between half-hourly eddy-covariance–based flux data with chamber-based flux data were calculated for control plots CC (after Spearman, data not normally distributed), for each measurement campaign separately. If a good agreement was found (*r* > 0.75), the average offset between daily sums of eddy-covariance flux and chamber flux data was calculated [see Appendix C (**Supplementary Table**, **Supplementary Figure 2–4**) in **Supplementary Material**]. In between campaigns, offsets were interpolated by the nearest-neighbor method. Subsequently, continuous time series of daily sums of ET and NEE for the measurement period were calculated by applying the interpolated offset to the eddy-covariance flux data and seasonal budgets derived. Chamber-based measurements included all treatments (CC, CD, NC, ND); therefore, we also applied this procedure to all treatments. Results of partitioned NEE (i.e., GPP and R_eco_) and ET (E and T) including the calculation of WUE_can_ were not inferred on the whole growing season. 

### Water Use Efficiencies

Partitioning NEE and ET allowed the calculation of water use efficiencies on both the canopy level and the ecosystem level. Water use efficiencies (WUE) were calculated from chamber-based daily flux sums, defined as canopy WUE_can_ (WUE_can_ = GPP/T) and ecosystem WUE_eco_ (WUE_eco_ = NEE/ET, with NEE defined as ≥0 for assimilating carbon). Standard deviations of WUE_can_ were high (±15%-47%) since standard deviations of WUE_can_ accounted for standard deviations of partitioned GPP and T. Ecosystem water use efficiencies WUE_eco_ for the whole growing season were calculated from daily flux sums based on the continuous and calibrated eddy-covariance flux data.

### Statistical Analysis 

Analysis focused on the growing season 2017 to study the long-term effects and adaptations to the treatments nitrogen addition and precipitation reduction. Results were presented as mean values ± 1 standard error (n = 4). All statistical analysis was conducted by comparing the single data of all plots over the whole growing season. For the LAI and species richness (in forbs), we performed linear mixed-effect models with analyses of variances (ANOVAs) to test for significant differences between treatments, with nitrogen addition and precipitation reduction as fixed effects (including interaction effects). To account for plot design and repeated measures, we added plot and date as random effects. Before analysis, residual-versus-fitted plots and quantile-quantile plots based on the model were plotted to validate the assumptions of homogenous variance and normal distribution of residuals of linear mixed-effects ANOVA models. Leaf area index and species count (forbs) were transformed by square root to meet criteria of ANOVA. Relative cover, absolute cover, and VWC data (which did not meet assumptions of ANOVA, even with transformation) were analyzed with beta regression models to test for the fixed-effects nitrogen addition and precipitation reduction on cover and VWC, with plot and date as random effects. Analysis on VWC was conducted on daily averages of VWC. VWC data were gap filled by using linear interpolation. For missing data of longer time periods (>10 days), VWC of the broken sensor was modeled by calculating the average offset between the respective broken sensor (mean of last 10 data points) and the mean VWC of other replicates (at the same time) and adding this offset to the mean VWC of other replicates for the missing data period. The effects over the whole growing season were tested over all depths together, and each depth separately. Additionally, the treatments effects on VWC within the distinct growing phases of the grassland were analyzed. Models for carbon and water fluxes were calculated with mean observations (n = 3) and standard deviations for models derived by error propagation. Results for partitioned water fluxes, partitioned carbon fluxes, and water use efficiencies (WUE_can_ and WUE_eco_) were presented as mean values ± 1 standard deviation (n = 3) and considered significantly different if standard deviations did not overlap. The relationship between vegetation parameters and daily (carbon/water) fluxes was assessed by linear mixed-effect models, accounting for repeated measurements (date as random effect). Here, the treatment means over the whole growing season at different time points (date) were compared. Residual-versus-fitted plots and quantile-quantile plots based on the model were plotted to validate the assumptions of homogenous variance and normal distribution of residuals of linear mixed-effects models. Probability values and correlations of determinations were derived based on the models. All analyses were conducted with R (version 3.6.0). Software packages used for models were *lme4* (Bates et al., 2019), *MuMIn* (Bartoń, 2019), *lmerTest* (Kuznetsova et al., 2019), and *glmmTMB* (Magnusson et al., 2019). 

## Results

### Environmental Conditions

**Figure 1:**
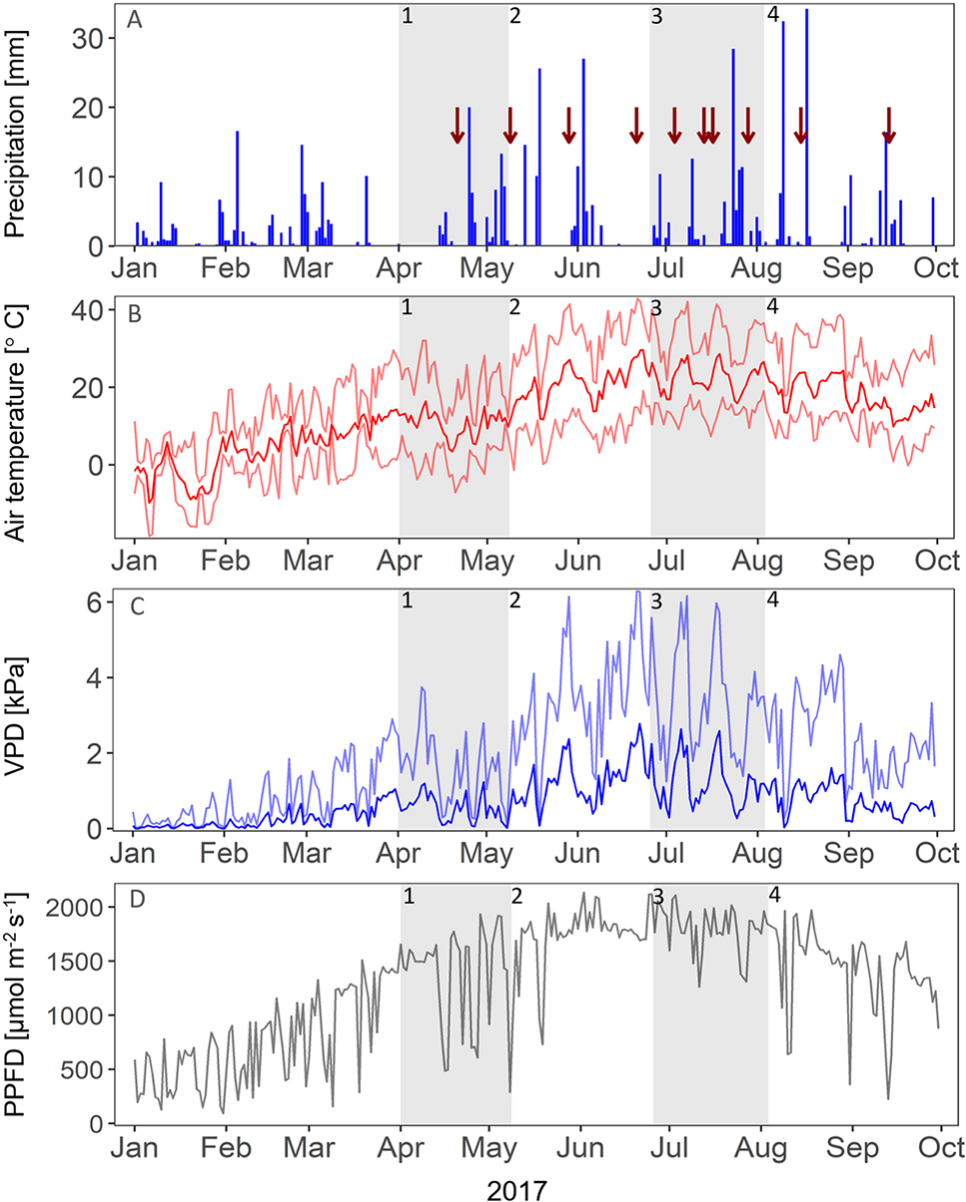
Environmental conditions in 2017. Red arrows indicate the dates of measuring campaigns. **(A)** Daily sum of precipitation [mm]. **(B)** Mean (dark red), minimum, and maximum (light red) air temperature [°C]. **(C)** Mean (dark blue) and maximum (light blue) vapor pressure deficit (VPD, in kPa). **(D)** Maximum daytime photosynthetic photon flux density (PPFD, μmol m-2 s-1). Gray panels indicate the different growth stages of the grassland (1-4).

The environmental parameters of the study site in 2017 followed the typical cool temperate climate, with spring starting from April, midsummer in June, and start of autumn in September (**Figure 1**). A late frost occurred in April. In the beginning of April, end of May, and end of June, multiple-day dry periods occurred with little to no rainfall. During the growing season, the mean air temperature (April-September) on the field site above canopy (1-m height) was 18.1 °C; 443.1-mm precipitation fell from April to September. When comparing the growing season 2017 to the long-term mean (1987-2016), we found that the growing season of 2017 was slightly cooler and drier (comparison of only DWD weather station data, approximately 700-m distance to field site). The mean air temperature from April to September was 16.7 °C, compared to the long-term mean (1987-2016, April-September) of 17.0 °C; 446.6-mm precipitation fell from April to September, which is below the long-term mean (1987-2016, April-September) of 527.2 mm (Deutscher Wetterdienst DWD, 2018). Drought-treated plots experienced approximately 282.7-mm rain from April to September (36.7% average exclusion by shelters from 446.6), which is 12% below the driest growing season on record (April-September 2003: 321.1 mm; DWD, Deutscher Wetterdienst and Climate Data Center, 2018). Heavy rain events (> 15 mm h^−1^) on the field site occurred in July and August, with, respectively, 28.4 and 34.2 mm (daily sum) on July 24 and August 18. Considering the seasonal dynamics of vegetation parameters and ecosystem fluxes, the growing season of 2017 could be divided in four distinct growth stages: season start in spring (stage 1), summer peak (stage 2), summer dieback (stage 3), and autumn peak (stage 4). This subdivision in growth stages will be further used to discuss our results. In the following, we will present our results regarding the impact of nitrogen addition and precipitation reduction on the studied grassland ecosystem, 2 years after the treatments have started.

### Treatment Effects on Vegetation

**Figure 2:**
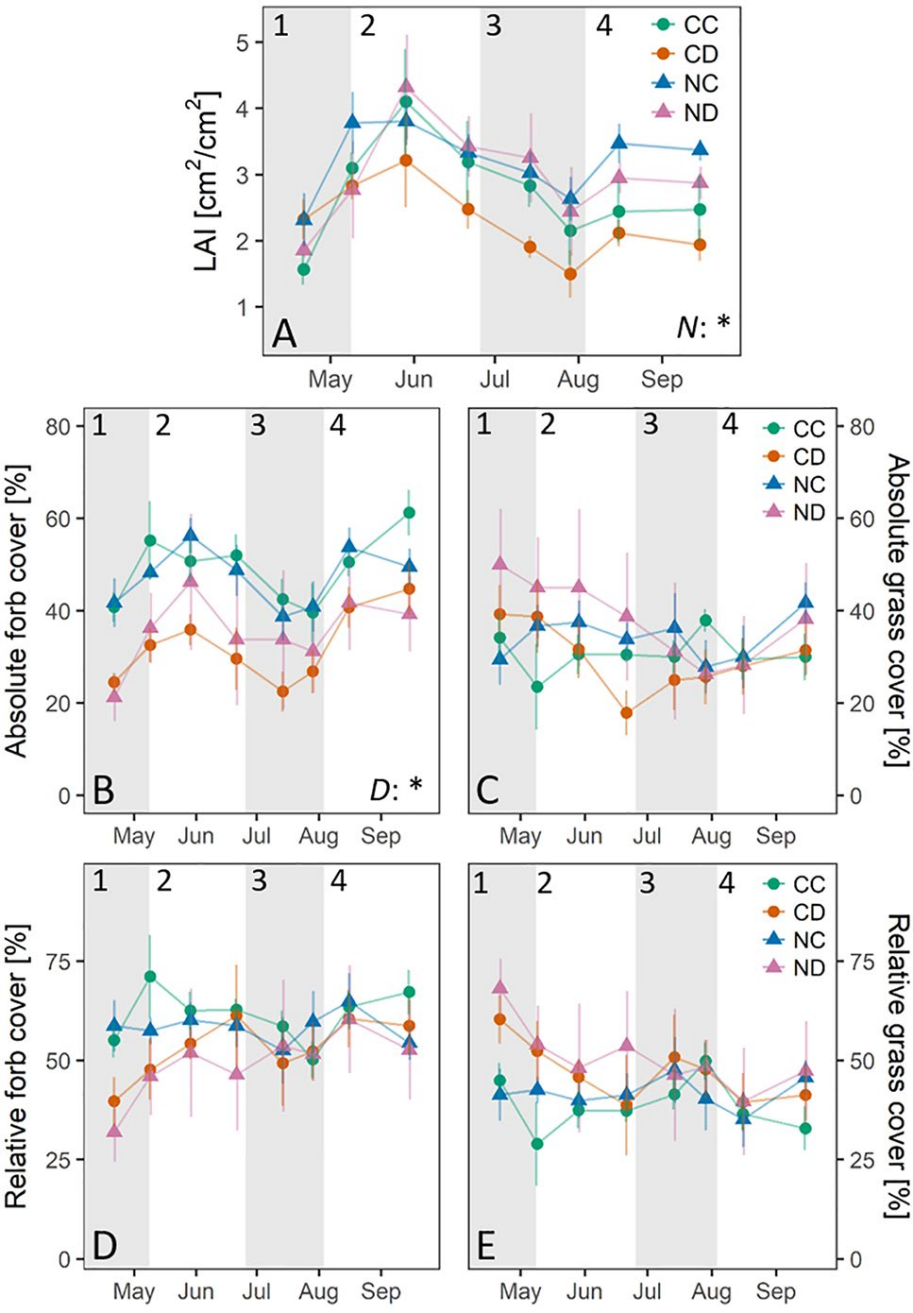
Leaf area index (LAI, in cm^2^ cm^-2^) per treatment. **(A)**. Absolute cover of functional groups: grasses. **(B)** and forbs **(C)** [%], per treatment. Relative cover of functional groups on total cover: grasses **(D)** and forbs **(E)** [%], per treatment. Treatments: CC (control, green), CD (precipitation reduction, orange), NC (nitrogen addition, blue), ND (precipitation reduction and nitrogen addition, rose). Reported values are mean ± 1 standard error. n = 4. Asterisks indicate significant effects of nitrogen (*N*) and/or drought (*D*). Significance level: *<0.05. Gray panels indicate the time frame of the different growth stages of the grassland (1-4).

The LAI of all treatments followed the typical spring-summer-autumn pattern of temperate grassland ecosystems (**Figure 2**). We could observe a season start in spring (stage 1), a first growth peak in early summer (stage 2), followed by strong dieback in late summer (stage 3), and a second growth peak in autumn (stage 4). Treatments had only a small impact on LAI: Nitrogen addition significantly increased LAI (*p* < 0.05), mainly in early summer (stages 1-2) and during the second growth peak. Precipitation reduction generally decreased LAI by 16% for CD (i.e., CD vs. CC, not significant, stage 3). Precipitation reduction, though, seemed to be an important driver for treatment differences and strong seasonal changes in forb and grass abundance (absolute and relative cover, **Figure 2**). Across the whole growing season, precipitation reduction strongly reduced the absolute forb cover (*p* < 0.05), of unfertilized plots CD by 34%, in combination with nitrogen addition by 25% (i.e., ND). At season start (stage 1), grass species dominated drought-treated plots CD (60%) and ND (68%), while the relative forb cover of CD and ND was lower relative to CC (CD: 40%, ND: 32%). During peak growth (stages 2 and 3), the relative forb and grass cover of CD and ND became more similar to CC and NC, which was related to an increasing forb growth and a strong decline in the absolute grass cover for both CD and ND plots. Nevertheless, absolute differences in forb cover remained strong. Grass cover strongly decreased on drought-treated plots between spring and summer (stages 1 and 3), which was the strongest for fertilized plots (for CD: −35%; for ND even: −48%). The combination of nitrogen addition and precipitation reduction seemed to promote the growth and dominance of grasses at season start; however, ND plots suffered from the strongest dieback during the dry summer season (stage 3).

**Figure 3:**
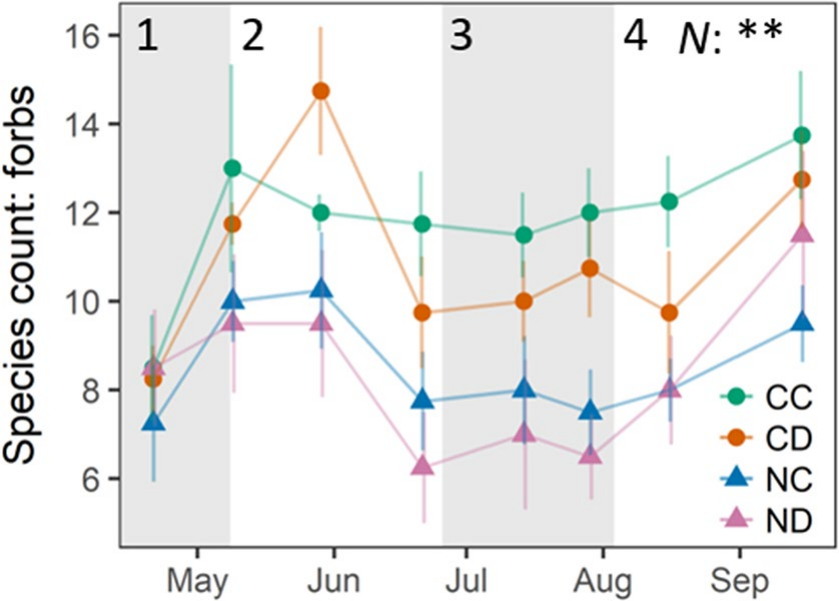
Number of present forb species, per treatment. Treatments: CC (control, green), CD (precipitation reduction, orange), NC (nitrogen addition, blue), ND (precipitation reduction and nitrogen addition, rose). Reported values are mean ± 1 standard error, n = 4. Asterisks indicate significant effects of nitrogen (N) and/or drought (D). Significance level: **<0.01. Gray panels indicate the different growth stages of the grassland community (1–4). See Appendix D (**Supplementary Table 2**), **Supplementary Material**, for a list of all forb species found on plots.

While precipitation reduction (i.e., CD vs. CC) only slightly affected species richness, nitrogen addition had a strong negative impact during and after the natural occurring summer drought (**Figure 3**). Nitrogen addition significantly decreased the amount of forb species present on NC plots by 33% and on ND plots by 36% (*p* < 0.01). Also, here the combined effect of precipitation reduction and nitrogen addition had the strongest (negative) effect on forb species richness. Forb diversity of the control treatment CC remained relatively stable over the whole growing season. No changes were found in the abundance of legumes.

### Treatment Effects on Soil Water

**Figure 4:**
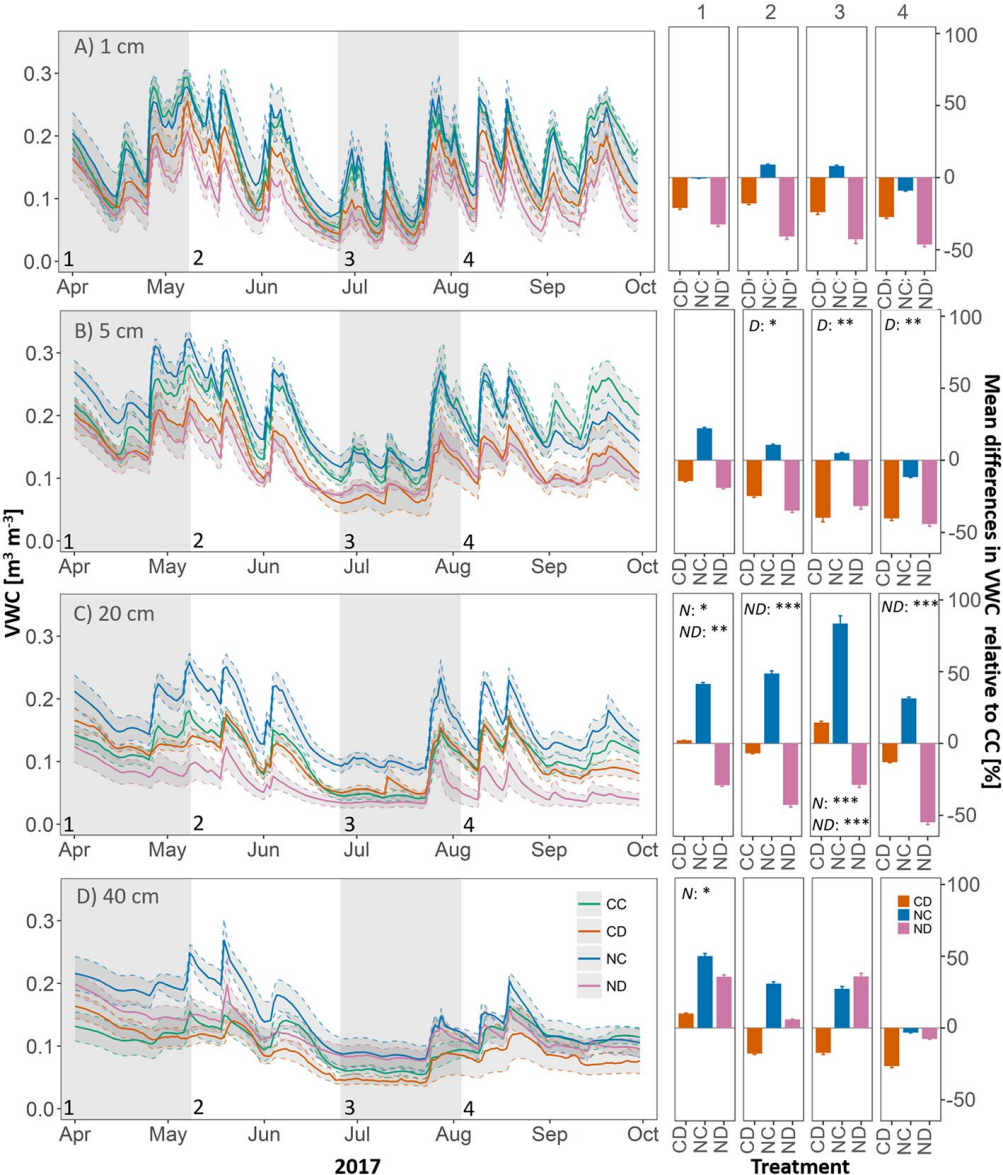
Left panels: Volumetric water content (VWC, in m^3^ m^-3^). Right panels: Mean differences in VWC relative to CC [%], during growth stages of the grassland (1-4). In soil depth **(A)** 1 cm, **(B)** 5 cm, **(C)** 20 cm, **(D)** 40 cm, per treatment. Gray panels and numbers indicate the time frame of the different growth stages of the grassland (1-4). Treatments: CD (precipitation reduction, orange), NC (nitrogen addition, blue), and ND (nitrogen addition and precipitation reduction, rose). Reported values are mean ± 1 standard error, n = 4. Standard errors of mean differences were derived by error propagation. Asterisks indicate significant effects of nitrogen (N), drought (D), and interactions between them (ND). Significance level: *<0.05, **<0.01, ***<0.001.

Over the whole growing season (April-September) and all depths, precipitation reduction significantly decreased VWC (*p* < 0.05, **Figure 4**), by 20% ± 0.3% for (CD vs. CC and 38% ± 0.5% for NC vs. ND). The effect of precipitation reduction was most pronounced in 5-cm depth (*p* = 0.014), with a mean reduction in VWC of up to 37% (ND vs. NC) and 31% (CD vs. CC). Nitrogen addition strongly affected VWC at 20 cm (*p* = 0.03), indicating a different water use and/or uptake due to changes in vegetation cover. While nitrogen addition alone (i.e., NC vs. CC) generally increased VWC by 46% ± 25% at 20-cm depth, in combination with drought it strongly reduced VWC at this depth (42% ± 23%). At this depth (only), strong interactions (*p* < 0.001) were found between nitrogen addition and reduced precipitation. Regarding the seasonal dynamics of VWC and treatment effects over the course of the growing season, we could observe a distinct dry down phase, which started middle of June and led to a long-term drop in VWC at 20- and 40-cm depths.

### Treatment Effects on Carbon and Water Fluxes

#### Partitioned Carbon and Water Fluxes

**Figure 5:**
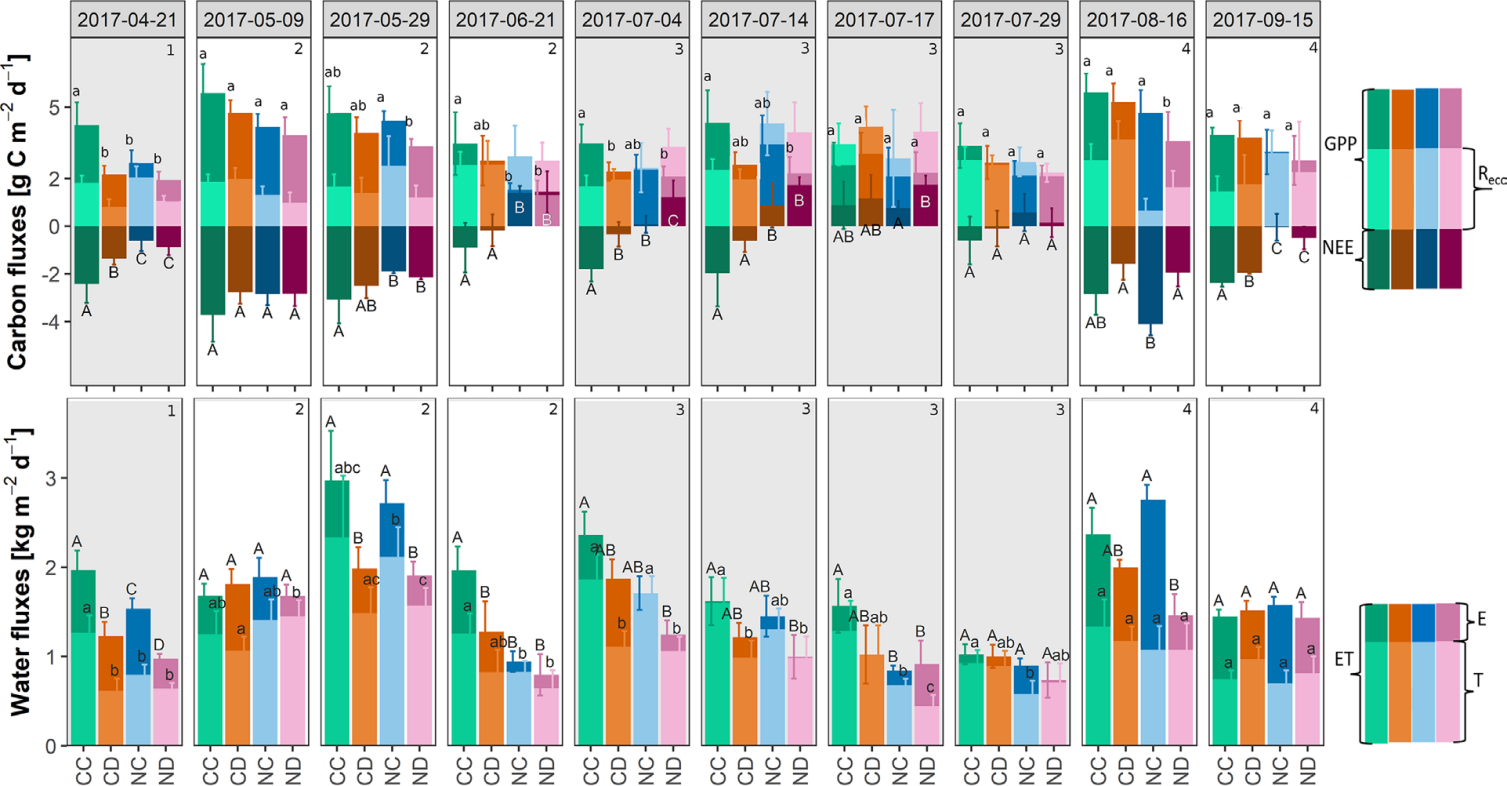
Top: Partitioned net ecosystem exchange (NEE): modeled daily rates of carbon fluxes during growing season 2017 [g C m^-2^ d^-1^], per treatment: gross primary production (GPP) and ecosystem respiration (R_eco_). Bottom: Partitioned evapotranspiration (ET): modeled daily rates of water fluxes during the growing season 2017 [kg H_2_O m^-2^ d^-1^], per treatment: soil evaporation E and transpiration T. Treatments: CC (control, green), CD (precipitation reduction, orange), NC (nitrogen addition, blue), and ND (nitrogen addition and precipitation reduction, rose). Method: best fit with locally weighted scatterplot smoothing (loess) ± standard deviation (68% confidence interval), n = 3. Standard deviations of E are not depicted. Columns that do not share letters are significantly different (see for E and R_eco_ in Appendix E (**Supplementary Table 3**), **Supplementary Material**). Gray panels and numbers indicate the different growth stages of the grassland (1-4).

Partitioning NEE and ET revealed the treatment effects on the single carbon and water fluxes (**Figure 5**). Precipitation reduction generally decreased GPP (CD vs. CC: -20% and ND vs. NC -17%) and increased *R*_eco_ (CD vs. CC: +7% and ND vs. NC: +36%, ≤ 3 campaigns significantly different for both GPP and *R*_eco_). Nitrogen addition in combination with reduced precipitation showed the highest negative impact on GPP, with 38% (i.e., ND vs. CC; significantly different for 5 out of 10 campaigns). At the end of stage 2, respiratory carbon release exceeded assimilation (positive NEE) on fertilized plots indicating the importance of processes in the soil.

Effects of precipitation reduction on T were small (≤ 3 campaigns significantly different), it generally decreased T (CD vs. CC: −27% and ND vs. NC −16%). Nitrogen addition in combination with reduced precipitation showed the highest negative impact on T, with 32% (i.e., ND vs. CC; significantly different for 5 of 10 campaigns).

#### Seasonal Budget of NEE and ET

**Figure 6:**
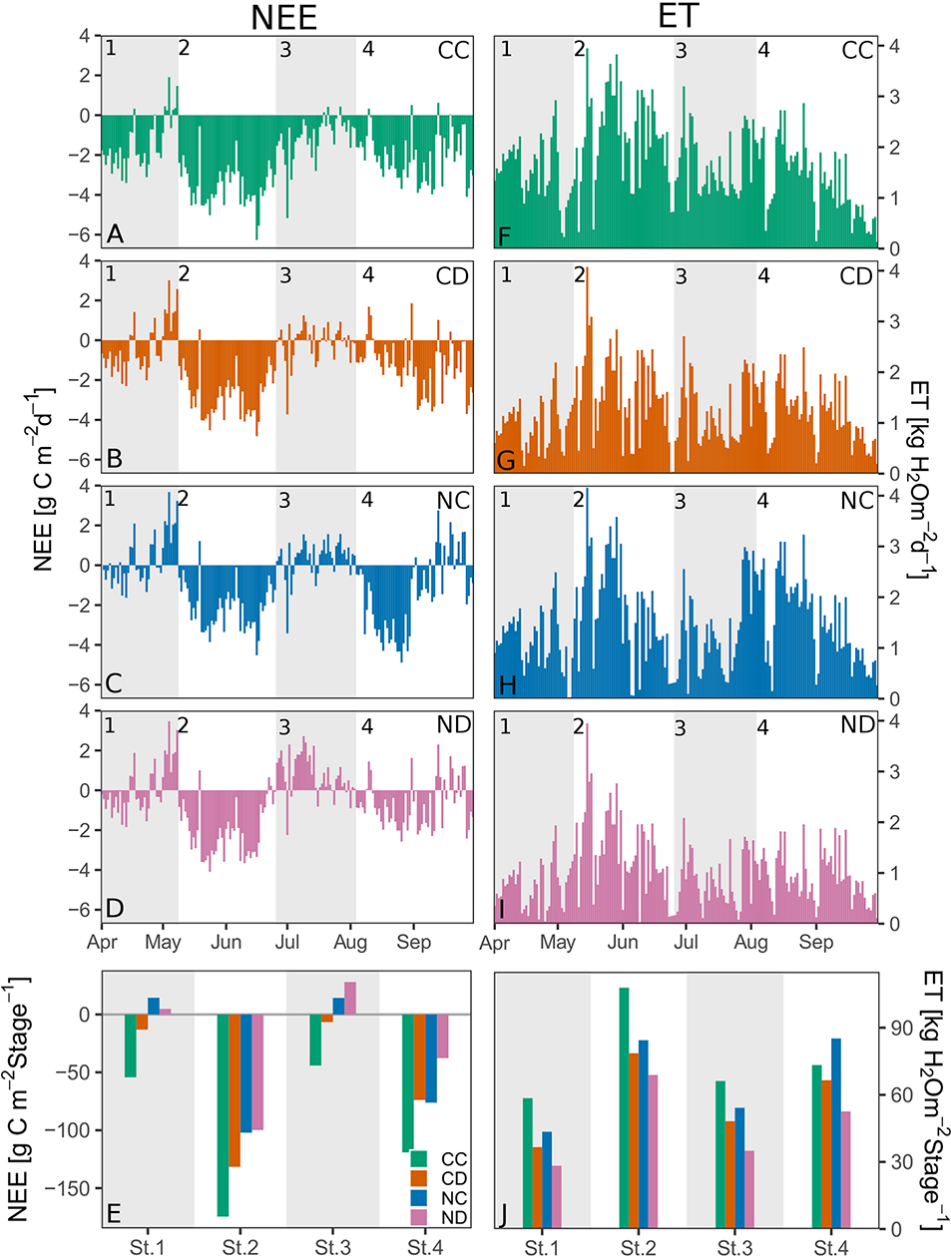
Left panels: Eddy-covariance–based net ecosystem exchange (NEE), per treatment. Daily **(A–D)** and stage **(E)** sums during growing season 2017 [g C m^−2^]. Right panels: Eddy-covariance–based evapotranspiration (ET), per treatment. Daily **(F–I)** and stage **(J)** sums during growing season 2017 [kg H_2_O m^−2^]. Treatments: CC (green, control), CD (orange, precipitation reduction), NC (blue, nitrogen addition), and ND (rose, precipitation reduction and nitrogen addition). Gray panels and numbers indicate the time frame of the different growth stages of the grassland (1-4).

Combining chamber-based measurements (**Figure 5**) with eddy-covariance–based data allowed the seasonal budgeting of NEE and ET (**Figure 6**). Summing up the water and carbon fluxes over the different growth stages, we found that during all growth stages, precipitation reduction generally reduced ET and NEE (i.e., CD vs. CC, and ND vs. NC); only in stage 3 was the absolute NEE of ND higher than of NC. Nitrogen addition (i.e., NC vs. CC, ND vs. CD) decreased ET and absolute NEE. Consequently, the combined effect of precipitation reduction and nitrogen addition (ND plots) had the strongest negative impact on ET and NEE.

Over the whole growing season (**Table 1**, period April-September), the control treatment CC functioned as the highest carbon sink, with about 389.6 g C m^−2^, followed by the treatments CD, NC, and ND (222.9, 157.3, 106.5 g C m^−2^, respectively). Nitrogen addition decreased the absolute value of NEE even stronger than precipitation reduction (60%; NC vs. CC; significantly different for 6 of 10 campaigns, **Figure 5**). In combination with reduced precipitation, the negative impact was the highest, with 73% (i.e., ND vs. CC; significantly different for 7 of 10 campaigns, **Figure 5**). Evapotranspiration was reduced by up to 40% as a consequence of both nitrogen addition and precipitation reduction, with ET sums for the growing season equal to 306.0, 229.9, 267.2, and 184.8 kg H_2_O m^−2^ for CC, CD, NC, and ND, respectively. The negative impact was the highest for the combined treatment, with 40% (i.e., ND vs. CC; significantly different for 7 of 10 campaigns, **Figure 5**).

**Table 1:** Sum over growing season 2017 (April-September), per treatment: evapotranspiration (ET) [kg H_2_O m^−^^2^], net ecosystem exchange (NEE) [g C m^−^^2^], ecosystem water use efficiency (WUE_eco_) (net ecosystem exchange [NEE]/evapotranspiration [ET]; in g C kg^−^^1^ H_2_O). Treatments: CC (control), CD (precipitation reduction), NC (nitrogen addition), and ND (nitrogen addition and precipitation reduction).

Treatment	NEE	ET	WUE_eco_
	(g C m^−2^)	(kg H_2_O m^−2^)	(g C kg^−1^ H_2_O)
**CC**	−389.6	306.0	1.27
**CD**	−222.9	229.9	0.97
**NC**	−157.3	267.2	0.59
**ND**	−106.5	184.8	0.58

#### Water Use Efficiencies

**Figure 7:**
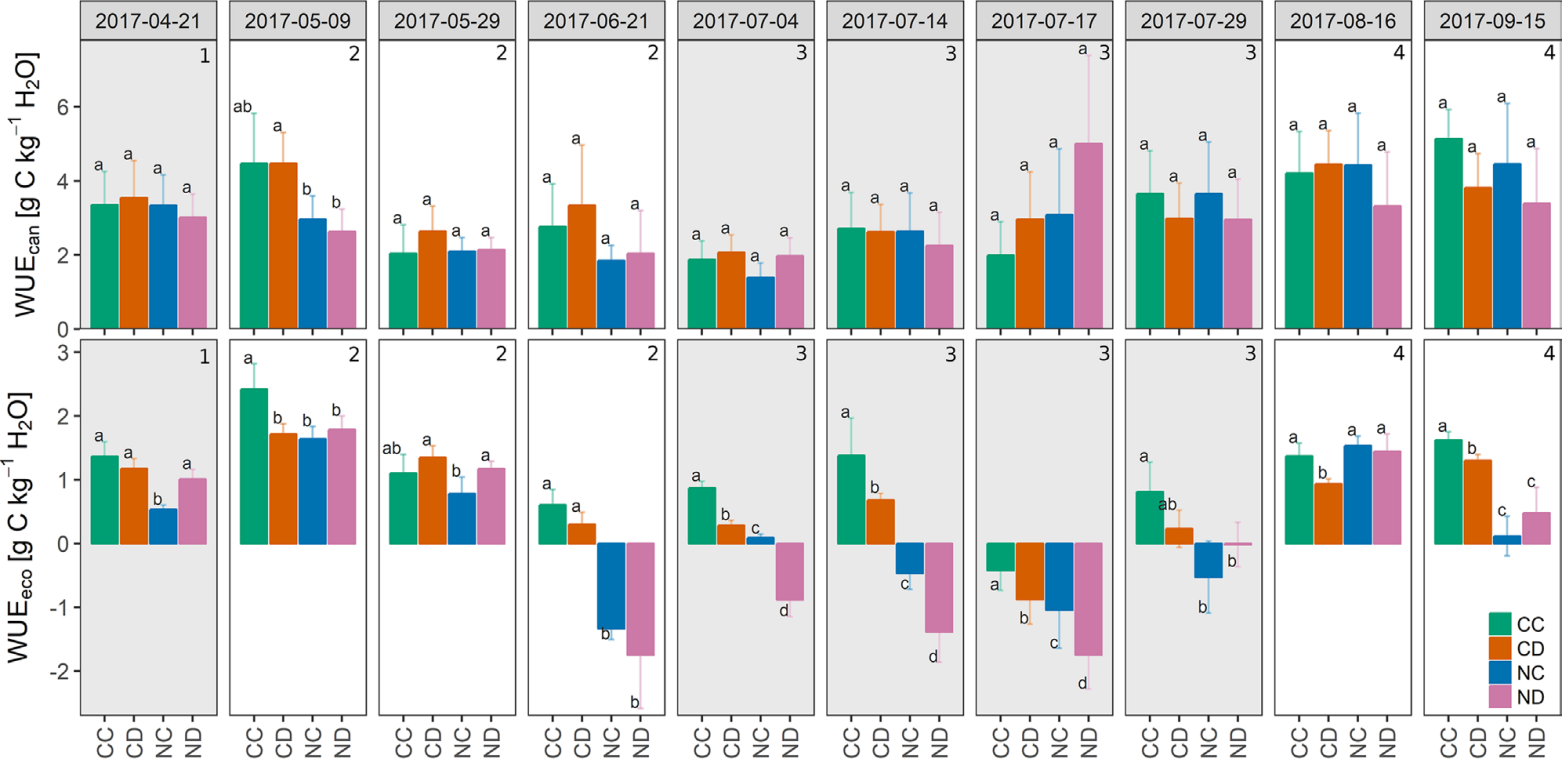
Top: Mean canopy water use efficiency WUE_can_ (gross primary production GPP/canopy transpiration T) during the growing season 2017, per treatment [g C kg^−1^ H_2_O]. Bottom: Ecosystem water use efficiency WUE_eco_ (net ecosystem exchange NEE/evapotranspiration ET), per treatment [g C kg^−1^ H_2_O]. Treatments: CC (control, green), CD (precipitation reduction, orange), NC (nitrogen addition, blue), ND (precipitation reduction and nitrogen addition, rose). Reported values are mean ± standard deviation, n = 3. Columns that do not share letters are significantly different. Gray panels and numbers indicate the different growth stages of the grassland (1-4).

Partitioning NEE and ET enabled us to calculate the water use efficiencies of the canopy WUE_can_ (= GPP/T, **Figure 7**). Neither precipitation reduction nor nitrogen addition (besides of measurement campaign “2017-05-09”) had a significant effect on WUE_can_. Water use efficiencies calculated at the ecosystem level WUE_eco_ (= NEE/ET see **Figure 7** and **Table 1**), however, show that nitrogen addition led to a less efficient use of water (−54%, NC vs. CC; significantly different for 8 out of 10 campaigns), and similarly strong in combination with precipitation reduction (−55%, ND vs. CC; significantly different for 7 of 10 campaigns), suggesting nitrogen addition as the main driver. At the end of stage 2, WUE_eco_ values became negative for fertilized plots since respiratory carbon release exceeded assimilation (as mentioned before). Precipitation reduction decreased WUE_eco_ by 23% (CD vs. CD; significantly different for 6 of 10 campaigns).

### Relating Ecosystem Fluxes to Vegetation and Environmental Dynamics 

Bringing together our results, we could observe that vegetation parameters (**Figures 2** and **3**) and ecosystem fluxes (**Figure 6**) of all treatments showed distinct strong seasonal variation following the typical spring-summer-autumn pattern of temperate grassland ecosystems: season start in spring (stage 1), summer peak (stage 2), summer dieback (stage 3), and autumn peak (stage 4). 

After a dry period in late winter/early spring (stage 1), fertilized plots (NC and ND) started with significantly smaller carbon fluxes (NEE, GPP) than control plots CC (**Figure 6**). A late frost event end of April harming the vegetation may have caused the carbon release at the end of stage 1. Drought-treated plots (CD and ND) showed significantly lower water fluxes (ET, E, T) than the control plots CC in stage 1. 

In spring and early summer (stage 2), in the peak growth stage of grassland species, fluxes were high for all treatments, with fluxes up to 6.3 g C m^−2^ and 4.2 kg H_2_O m^−2^ (ET) per day. During this time, species richness and LAI were high for all treatments (**Figures 2** and **3**). During a long dry period of 2 weeks with less than 3.5-mm precipitation in total and mean air temperatures above 20 °C (stage 2, **Figure 1**), soil moisture drastically dropped in all depths (< 0.12 m^3^ m^−3^, **Figure 4**). Consequently, both transpiration and evaporation rates strongly declined, in particular of fertilized plots (NC and ND, **Figures 5** and **6**).

A dieback of the vegetation, especially for NC and ND, strongly decreased GPP and strongly increased R_eco_ (stage 3; see **Figures 5** and **6**). Fertilized plots even became carbon sources (positive NEE). This trend continued throughout July, with soil moistures in 20 and 40 cm depth remaining low. At mid-July, all treatments became carbon sources. At the end of July, a longer rain period led to a slow recovery of the grassland ecosystem, whereas especially NC seemed to recover fast with high absolute NEE values (**Figure 5**).

A second peak of NEE and ET occurred in late summer/early autumn (stage 4), especially for NC, with fluxes up to 4.9 g C m^−2^ and 2.9 kg H_2_O m^−2^ per day (**Figure 6**). This recovery caused a second vegetation peak in all treatments, which correlated with an increase in LAI (**Figure 2**). However, drought-treated plots (i.e., CD and ND) seemed to recover slower with a 38% (50%) higher absolute NEE for CD versus CC (ND vs. NC). At the end of the growing season (stage 4), fertilized plots (NC and ND) became carbon sources again (**Figures 5** and **6**). With more frequent rain events and increasing soil water content, water fluxes significantly increased from August onward (end of stages 3 and 4), with no/small differences between treatments. In general, transpiration dominated the water fluxes of the grassland ecosystem. The fraction of transpiration to ET *ft* increased with time, corresponding to the increase in primary production and vegetation cover.

**Figure 8:**
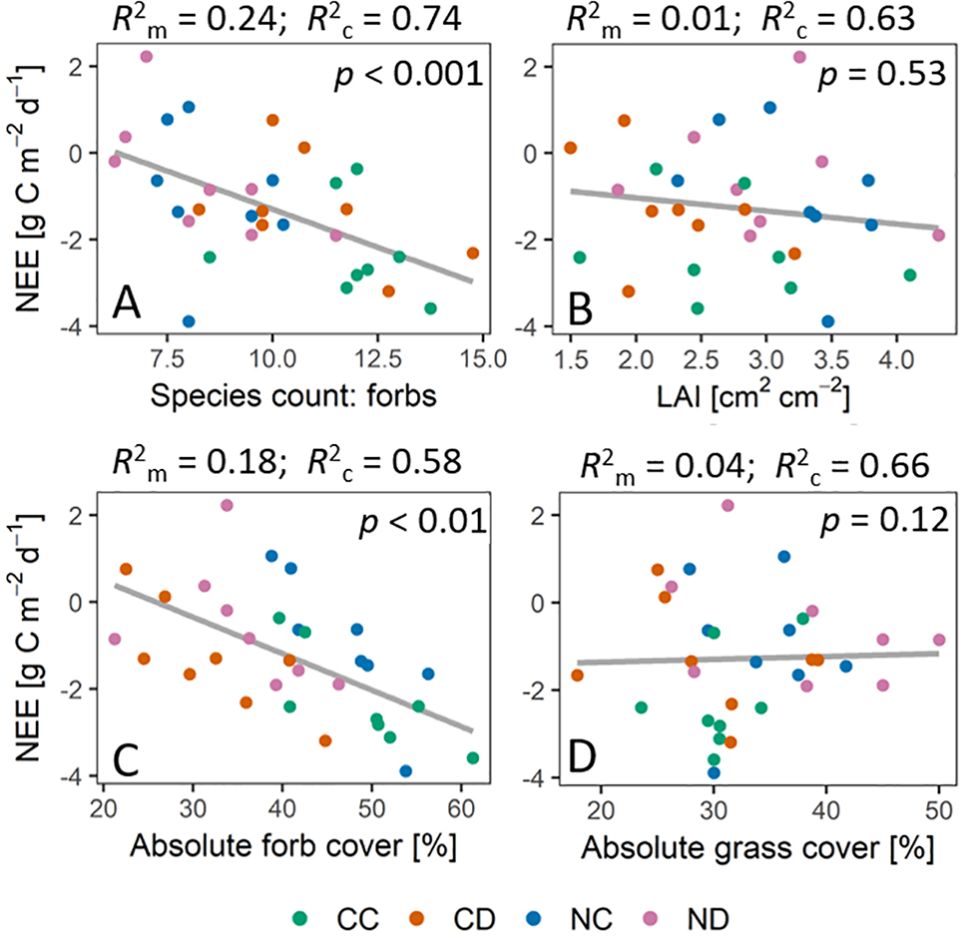
Relationship between net ecosystem exchange [NEE] and vegetation parameters during the growing season 2017, that is, daily sums of NEE [g C m^−2^ d^−1^] plotted against vegetation parameters (treatment means) at the same time point of measurements. Vegetation parameters: **(A)** Forb species richness. **(B)** LAI [cm^2^ cm^-2^] **(C)** Absolute forb cover [%]. **(D)** Absolute grass cover [%]. p values indicate significant impact of respective vegetation parameters on NEE. *R* ^2^_m_ is the marginal coefficient of determination which describes the proportion of variance explained by the fixed factor (vegetation parameter). *R* ^2^_c_ is the conditional coefficient of determination, which describes the proportion of variance explained by both the fixed effect (vegetation parameter) and the random effect (date). Gray lines depict the linear regression lines based on the models. Treatments: CC (control, green), CD (precipitation reduction, orange), NC (nitrogen addition, blue), ND (precipitation reduction and nitrogen addition, rose).

Excluding the strong seasonal impact on ecosystem fluxes (by applying linear mixed-effect models), we could find a significant impact of forb species richness (*p* < 0.001) and absolute forb cover (*p* < 0.01) on NEE (**Figure 8**). Forb species richness could explain the dynamics in NEE by 24% (*R^2^_m_*). Accounting for (random) seasonal changes, forb species richness explained 74% (*R^2^_c_*) of the dynamics in NEE. Evapotranspiration was strongly affected by the absolute forb cover (*p* < 0.01, *R* ^2^_m_ = 0.11, *R* ^2^_c_ = 0.81) and less by forb species richness (*p* < 0.1, *R* ^2^_m_ = 0.04, *R* ^2^_c_ = 0.74). No significant impact of LAI was found on neither NEE (*p* = 0.53) nor ET (*p* = 0.46).

## Discussion

Temperate grasslands strongly depend on precipitation as a main water resource (Leitinger et al., 2015). A projected increase in drought events for Central Europe (Kovats et al., 2014; Spinoni et al., 2018) will largely affect these ecosystems. Many studies show that decreased precipitation, which results in less available soil moisture, clearly affects ET fluxes (e.g., Wever et al., 2002), and limits plant productivity in grasslands (e.g., Tilman and El Haddi, 1992; Tilman and Downing, 1994; Kahmen et al., 2005b). Our results, however, show that also changes in nutrient availability can substantially affect carbon and water cycling of grassland communities through vegetation/biodiversity effects (**Figure 8**).

One main observation of our research is that the studied grassland ecosystem, substantially altered in species composition by nitrogen addition, lost its function as a carbon sink (positive NEE) during the dry summer period (stage 3, **Figures 5** and **6**). Summed up over the whole growing season (April-September, **Table 1**), the sequestration of carbon was reduced by more than 60% as a consequence of nitrogen addition. Extreme drought (i.e., reduced precipitation) reduced carbon sequestration by 43%, and its negative impact was not counteracted by nitrogen addition (treatment ND relative to CC: −73% in NEE). Precipitation reduction seemed to promote the growth and dominance of grass species at season start (**Figure 2**, stages 1 and 2). However, during the dry summer months under additional natural drought conditions, grass species suffered from a strong (above-ground) dieback (−35% to 48% of absolute grass cover on CD and ND plots). Concurrently, nitrogen loading caused a significant loss in forb species (−25%, April-September) and enhanced the negative impact of simulated and natural drought stress on the grassland ecosystem by weakening the community’s stress response. Dynamics in NEE and ET could not be explained by changes in LAI (*p* = 0.5) as observed in other studies (Hu et al., 2009; Schmitt et al., 2010; Lü et al., 2014). 

In many terrestrial ecosystems, net primary production is limited by nitrogen availability (LeBauer and Treseder, 2008). For temperate grasslands, some studies found an increase in grassland (above-ground) productivity with nitrogen addition (e.g., Wedin and Tilman, 1996) or decrease with nitrogen cessation (e.g., Pallett et al., 2016). In our system, GPP and absolute NEE in early summer (stage 2) were, in fact, high for fertilized plots NC and NC. The highest GPP and absolute NEE, though, were found for the control treatment CC. In addition, fertilized plots NC and ND became carbon sources in the summer months (stage 3, **Figures 5** and **6**), while the treatments CC and CD with higher species richness (despite lower LAI in case of CD, **Figures 2** and **3**) remained carbon sinks, suggesting the importance of species and functional diversity for sustainable ecosystem functioning. Furthermore, nitrogen addition might have altered processes in the soil causing higher R_eco_ rates of fertilized plots. One reason could be an accelerated decomposition of dead biomass (Mack et al., 2004).

Harpole and Tilman (2007) found in a similar experiment (nitrogen and water addition in a Mediterranean grassland) that only unfertilized plots with added water maintained net carbon uptake at the end of the experiment. Fertilized plots might have not been able to maintain plant productivity later in the season due to stronger water depletion at season start (high productivity) and earlier senescence of leaves. However, in our study, only the soil moisture of ND plots was stronger depleted (**Figure 4**). 

Nitrogen loading is known to significantly decrease species richness in temperate grasslands (Wedin and Tilman, 1996; Stevens, 2004; Stevens et al., 2006), especially forb diversity (Stevens et al., 2006), and fertilization clearly decreases species richness (Socher et al., 2013). Higher species richness, though, can sometimes lead to higher productivity of grassland systems (Pfisterer and Schmid, 2002; Spehn et al., 2005; van Ruijven and Berendse, 2005), also under drought conditions (Pfisterer and Schmid, 2002). In more diverse systems, species are in need to find and occupy certain niches due to higher competition, which allows a system to support more species of different functional groups than only few dominant species that deplete specific resources intensively. van Ruijven and Berendse (2005) found that increased efficiency in nutrient usage in more diverse grasslands led to higher plant productivity (i.e., aboveground biomass). More diverse grassland communities were further found to have higher nitrogen use efficiencies (Kleinebecker et al., 2014). Vice versa, nitrogen addition might lead to an inefficient nutrient use (Lü et al., 2014). Here, we could observe a significant decrease in forb species due to nitrogen addition (−25%, in the dry summer months even up to −33%), which might explain the lower net primary production of fertilized plots, especially in the dry summer period under additional (natural) drought stress. Deep rooting forb species were, generally, found to be more drought resistant and to control the biomass resistance during summer drought, while shallow rooting grass species rather determined the biomass recovery of grassland communities after drought (Stampfli et al., 2018; Mackie et al., 2019). By the competitive exclusion of forb species (Tilman, 1997), nitrogen addition likely decreased the biomass resistance and plant productivity of the studied grassland during the summer drought. Long-term reduced precipitation seemed to additionally negatively affect forb species (**Figure 3**; absolute forb cover); that is, nitrogen loading in combination with long-term extreme drought exerted a strong negative impact on forb species richness and cover. Long-term extreme drought also seemed to have altered the temporal appearance of the functional groups. Precipitation reduction seemed to promote the growth and dominance of grass species at season start (**Figure 2**, stages 1 and 2). These altered temporal dynamics could indicate adaptions of the grassland community to long-term extreme drought by species asynchrony and temporal diversification in growth. Grassland communities were found to stabilize their plant productivity during disturbances by species asynchrony (Isbell et al., 2009; Hector et al., 2010; Hautier et al., 2014). This mechanism of species asynchrony, however, can be disturbed by nitrogen addition (Hautier et al., 2014), decreasing the stability of community productivity and weakening the community’s stress response to droughts.

Increased nitrogen availability and nitrogen efficiency can lead to increased water use efficiencies of plant species (Singh et al., 1975; Taylor et al., 1991). In this study, we found significantly higher water contents in fertilized plots (only NC with ambient rain, **Figure 4**), indicating a different water use/uptake by the fertilized grassland community. However, we could not find increased water use efficiencies for fertilized plots. In fact, the higher water contents of NC plots were likely related due to the large aboveground and probably also belowground biomass loss, which led to a lower water use. Moreover, a substantially altered species composition, caused by nitrogen addition, could also cause shifts in the root water uptake patterns due to the different root functional traits of grasses and forbs. Higher water contents, though, were not observed for fertilized and drought-treated plots (ND). Long-term reduced precipitation might have forced the grassland community to access deeper soil water (Garwood and Sinclair, 1979; Sharp and Davies, 1979).

Higher water use efficiencies of unfertilized plots (**Table 1** and **Figure 7**), in turn, might indicate that a higher species richness increases the efficiency of water use by the community and made the community more resistant to drought events. In a diverse grassland system, with generally shallow-rooting grass species and deep-rooting forb species, water resources might be more efficiently used by vertical niching (Hoekstra et al., 2014). Belowground production was found to be correlated to plant diversity, which likely enabled diverse grassland to maintain their ecosystem functions during drought (Kahmen et al., 2005b). Nitrogen addition, though, significantly reduced forb species richness in our grassland system and subsequently may have reduced the water use efficiency of the community. A less efficient water use in communities with higher nitrogen loading would reduce the water availability and might enhance the negative impact of drought stress on the community.

On the other hand, water availability is a key factor of nutrient availability for plants. It controls the transport of nutrients and may affect the transformation processes of nitrogen by microbiological activities. Under drought conditions, nitrogen uptake by plants was found to decline (Homyak et al., 2017), and microbial activities and thus nitrogen transformations decrease (e.g., Stark and Firestone, 1995). However, we could not find that nitrogen addition facilitated the negative impact of drought on NEE when comparing ND to CD. The response of a community largely depends on the ecohydrological and ecophysiological response of its individual species and functional groups. Different plant functional groups of grassland communities respond differently to nitrogen addition (You et al., 2017). While, on a global scale, the aboveground biomass of grasses significantly increased due to nitrogen addition, the aboveground biomass of forbs has not significantly changed (meta-analysis by You et al., 2017). Underlying mechanisms could be the advantage of grasses toward forbs in the competition for light (Hautier et al., 2009), higher tolerance to soil acidification (Tian et al., 2016) or higher root density in the upper soil layers where nitrogen addition took place (Huang et al., 2008). While grasses might be favored by nitrogen addition, drought stress may strongly hamper their growth (Gilgen and Buchmann, 2009; Grime et al., 2000; Signarbieux and Feller, 2008). Due to their shallow root systems, grasses are more susceptible to drought stress than forbs. In this study, the grass cover under reduced precipitation (i.e., CD and ND) strongly decreased during the dry summer months (−35-48%), whereas in combination with nitrogen addition (i.e., ND) the negative impact of drought stress was the strongest. At the same time, lower values of WUE_eco_ of fertilized plots NC and ND (−54%, **Table 1**) indicate a less efficient use of water on the ecosystem level as a consequence of nitrogen addition, which probably further enhanced the stress impact of drought. 

Reduced precipitation clearly decreased soil water content, especially in the depths of 5 and 20 cm, which are the most relevant depths for shallow rooting plants. The effect was more pronounced for fertilized plots, which might indicate that an altered species community due to nitrogen addition led to different water use and also uptake in different depths, maybe related to different root distributions. An increased nitrogen availability and/or nitrogen efficiency could allow species to allocate roots to deeper depths. The ecophysiological and ecohydrological response of plant species to water and nitrogen availability can largely affect their root system, which in turn can have complex feedbacks on the grassland system. Roots can allocate water and nutrients upward and function as pathways for water infiltration (e.g., Feddes et al., 1976, Meek et al., 1992, Bhark and Small, 2003, Dubbert et al., 2014). The response of a system to an altered (biotic and abiotic) environment changes with time and the long-term response of the system may still undergo dynamic development (van Ruijven and Berendse, 2005). Here, 2 years after the experiment started, we found that the studied grassland was substantially altered in its species composition due to nitrogen loading, and its function as carbon storage was strongly reduced. This loss in carbon sequestration potential was closely linked to a loss in biodiversity and a less efficient use of the resource water, which had strong cascading effects on the carbon and water fluxes of the system. A more decreased soil water pool may further enhance the negative impact on the nitrogen-loaded grassland community and decrease its resilience to cope with extreme drought. Our results suggest that, in face of climate change and increasing drought events, high species and functional diversity are crucial in order to preserve the carbon sequestration potential in temperate grasslands. Therefore, preserving high diversity of seminatural grasslands is not just pivotal for its intrinsic value but also to strengthen the resistance of systems against extreme events such as droughts. Future management practices should focus on reducing human caused nitrogen input into ecosystems and fight other threats to biodiversity loss. 

## Data Availability

The datasets generated for this study are available on request to the corresponding author.

## Author Contributions

AK, CW, YR, and MD planned and designed the experiment. AK, AP, CW, and MD set up the experiment. AK collected and analyzed the data and wrote the manuscript. MG and EK helped collecting and analyzing the data. AP processed the eddy-covariance data. All authors reviewed the manuscript.

## Funding

Funding was provided by the DFG (DU1688/1-1), the “Innovationsfond Forschung” (2100095601) and “Landesgraduiertenförderung” of the federal state of Baden-Württemberg. The article processing charge was funded by the German Research Foundation (DFG) and the University of Freiburg in the funding program Open Access Publishing.

## Conflict of Interest Statement

The authors declare that the research was conducted in the absence of any personal, commercial, or financial relationships that could be construed as a potential conflict of interest.
