# Supplementary material for: Nitrogen Loading Enhances Stress Impact of Drought on a Semi-natural Temperate Grassland
Source: Front Plant Sci. 2019 Aug 30;10:1051. doi: 10.3389/fpls.2019.01051 (PMC6730263; doi:10.3389/fpls.2019.01051)
Supplement: Supplementary file 1 [file Table_1.docx]

Supplementary Material

**Appendix**

### Appendix A: Methods: Isotopic signatures of evapotranspiration, leaf and soil samples

The water oxygen stable isotopic signature is reported here as δ-notation, relative to Vienna Standard Mean Ocean Water (V-SMOW; Gonfiantini, 1978: δ^18^O = [(*R_sample_* − *R_V_*_−_*_SMOW_*)/*R_V_*_−_*_SMOW_*] and are expressed in ‰.

# Evapotranspiration

Isotopic signatures of evapotranspiration were measured by coupling a cavity ring-down spectrometer (*CRDS*, L2120-i, Picarro Inc., Santa Clara, CA, USA) to gas exchange chambers. Isotopic signatures of evapotranspiration (*δ_ET_*) were determined by mass balance:

$\begin{aligned} \delta_{\text{ET}}=\frac{u_{\text{out}}w_{\text{out}}\delta_{\text{out}}-u_{\text{in}}w_{\text{in}}\delta_{\text{in}}}{u_{\text{out}}w_{\text{out}}-u_{\text{in}}w_{\text{in}}}= \\ \frac{w_{\text{out}}\delta_{\text{out}}-w_{\text{in}}\delta_{\text{in}}}{w_{\text{out}}-w_{\text{in}}}-\frac{w_{\text{in}}w_{\text{out}}\left( \delta_{\text{out}}-\delta_{\text{in}} \right)}{w_{\text{out}}-w_{\text{in}}} \end{aligned}$ (A1)

where *u* is the flow rate [mol (air) s^−1^], *w* the mole fraction [mol (H_2_O) mol (air)^−1^] and *δ* the isotope ratio of air. Subscripts denote the incoming (in) and outgoing (out) air stream of the gas chamber. The second term in Eq. 1 corrects for the air flow in the chamber due to addition of water by evapotranspiration *ET*. *δ, w* and *u* were measured by alternately measuring the atmospheric vapor (reference, incoming air stream) and mixed signal of *ET* and atmosphere (sample, outgoing air stream) with the CRDS.

# Soil evaporation and transpiration

In order to model the isotopic signature of transpiration and soil evaporation (see Appendix B: Isotope Theory), soil and leaf samples were collected for isotopic analysis parallel to gas chamber measurements. Soil (n = 3 per treatment, in total 12) and leaf samples (n = 3 per treatment, mixed leaf samples, in total 12) were collected in the afternoon (14:00-16:00) during flux measurements (assuming steady state of transpiration). All samples were taken in close vicinity to the metallic rings for chamber measurements. Leaf samples were taken from vegetation outside of the rings resampling the vegetation present in the rings. Major veins of herbaceous species were removed in order to sample solely mesophyll water. Soil and leaf water samples were immediately sealed, stored at -20°C and later extracted on a custom-built vacuum line by cryogenic distillation. Samples were heated at approximately 95 ºC for 90 min under vacuum of 0.8 Pa and water vapor was trapped in liquid N_2_ cooled water traps. Extracted water samples were stored in sealed glass vials at 4 ºC until analysis. Samples were measured with laser spectroscopy (Picarro L2130i, *CRDS*, Santa Clara, CA, USA) by liquid water injection into the vaporizer of the analyzer. Within every batch of 105 samples three replicates of three different laboratory standards plus a running standard after each 10 samples were analyzed for δ^18^O calibration. Laboratory standards were calibrated against V-SMOW, SLAP, and GISP (IAEA, Vienna). Analytical precision was 0.13 ± 0.17 ‰ for δ^18^O.

Results were post-processed with the Picarro software PostChemCorrect^TM^ to detect organic contamination. A certain amount of samples contaminated with organic compounds were analyzed by isotope-ratio mass spectrometry (Isoprime IRMS, Elementar, [Langenselbold](https://www.google.com/search?client=firefox-b-ab&q=Langenselbold&stick=H4sIAAAAAAAAAOPgE-LWz9U3MDQsNDWsSFbiBHFSigstcrQ0Msqt9JPzc3JSk0sy8_P084vSE_MyqxJBnGKr9MSiosxioHBGIQBw4zKGRAAAAA&sa=X&ved=2ahUKEwj-_fPvovfeAhXCjKQKHcxYAzYQmxMoATAUegQIBRAN), Germany), after headspace equilibration for 24 hours at 20 °C, connected via open split to a µgas auto sampler (Elementar, [Langenselbold](https://www.google.com/search?client=firefox-b-ab&q=Langenselbold&stick=H4sIAAAAAAAAAOPgE-LWz9U3MDQsNDWsSFbiBHFSigstcrQ0Msqt9JPzc3JSk0sy8_P084vSE_MyqxJBnGKr9MSiosxioHBGIQBw4zKGRAAAAA&sa=X&ved=2ahUKEwj-_fPvovfeAhXCjKQKHcxYAzYQmxMoATAUegQIBRAN), Germany). Results showed good agreement between samples measured by laser spectroscopy and isotope-ratio mass spectrometry.

**Appendix B: Isotope theory**

# Soil evaporation

The isotope ratios of soil evaporation (*R_E_*) were calculated using the Craig and Gordon model

(Craig & Gordon, 1965):

$R_{E}=\frac{\frac{R_{e}}{\alpha^{+}}-R_{v}h}{\alpha_{k}(1-h)}$ (A2)

where *R_e_* is the isotope ratio of bulk soil water at the evaporating site and *R_v_* the isotope ratio of atmospheric water vapor. The highest value of isotopic ratio of bulk soil water alongside the soil profile was chosen for *R_e_* and its respective temperature (*T_e_*) and soil water content (*x_e_*) taken for calculations. α*^+^* (α*^+^*>1) is the water vapor equilibrium fractionation factor and was calculated after Majoube (1971), using *T_e_*. *h* is the relative air humidity normalized to air temperature and *T_e_*. α*_k_* the kinetic fractionation factor (α*_k_* >1) and is defined as (Stewart, 1975):

$$\alpha_{k}=\left( \frac{D_{v}}{D_{v}^{i}} \right)^{nk}(A3)$$

with $\frac{D_{v}}{D_{v}^{i}}$ as the molecular diffusion coefficient, *D_v_* as diffusivity of H_2_O in air and $D_{v}^{i}$ as the diffusivity of H_2_^18^O in air. Here, we chose $\frac{D_{v}}{D_{v}^{i}}$ = 1.028 after Merlivat (1978) and Luz, Barkan, Yam, & Shemesh (2009). The exponent *nk* was calculated after Mathieu & Bariac (1996), using the soil moisture content on the evaporative front:

$$nk=\frac{\left( \theta_{e}-\theta_{r} \right)n_{a}+\left( \theta_{sat}-\theta_{e} \right)n_{s}}{(\theta_{sat}-\theta_{r})} (A4)$$

With *θ_r_*, *θ_sat_*, and *θ_e_* being the residual, the saturated volumetric soil water content, and the volumetric soil water content at the evaporative site. The residual soil water content found in the top layers (1 cm and 5 cm, year 2016-2017) was 0.035-0.11 m^3^ m^-3^, the saturated was 0.19-0.32 m^3^ m^-3^ (depending on treatment), *n_a_* and *n_s_* are 0.5 and 1.0 respectively, following Mathieu & Bariac (1996). For more details about calculating α*_k_* see Braud, Bariac, Gaudet, & Vauclin, (2005) and Dubbert et al. (2013).

# Transpiration

The isotope ratios of transpiration are derived from leaf water samples following the approach by Piayda, Dubbert, Siegwolf, Cuntz, & Werner (2017). Leaf water at the evaporative front (*R_e_*) was sampled during assumed steady state of transpiration in the early afternoon. Major veins from herbaceous species were removed and, therefore, the Péclet effect was not considered in this study. For modelling the isotopic signature of transpiration in the months August, the results of leaf water of the campaign two weeks before had to be taken.

The isotope ratios of xylem can be calculated using the Craig and Gordon model (Eq. A2), assuming that transpiration is in steady state in the early afternoon and that the isotope ratios of transpiration equal the isotope ratios of xylem. Here, *R_e_* is the isotope ratio of leaf water at the evaporating site and *h* the relative air humidity normalized to the leaf temperature.

The leaf water at the evaporative site in steady state *R_C_* can be calculated using the results of (Eq. A3) (*R_E_*, isotope ratio of xylem in steady state).

$R_{C}=\alpha^{+}\alpha_{k}(1-h)R_{E}+\alpha^{+}hR_{v}$ (A5)

The isotope ratios at the evaporative site in non-steady state *R_e_* can be calculated with an iterative model over time *dt* if leaf water *V_m_* (mol (H_2_O) m^−2^) is assumed constant (Cuntz, Ogée, Farquhar, Peylin, & Cernusak, 2007; Dongmann, Nürnberg, Förstel, & Wagener, 1974; Farquhar & Cernusak, 2005). *g_t_* is the leaf conductance for water vapor/canopy conductance of water vapor (mmol m^-2^ s^-1^) and *w_i_* the vapor saturation at leaf temperature (mol(H_2_O) mol(air)^−1^) (Eq. A4). As a starting point, 4 hours before sunrise was chosen, the starting value for *R_C_* was the isotope ratios of xylem, derived from Eq. A1. *R_e_* at a time *t* + *dt* is calculated from *R_e_* at an earlier time *t.*

(A6)

As a final step, the isotope ratio of transpiration in non-steady state *R_E_* can be derived using the Craig and Gordon model (Eq. A1).

# Partitioning evapotranspiration: example of month May


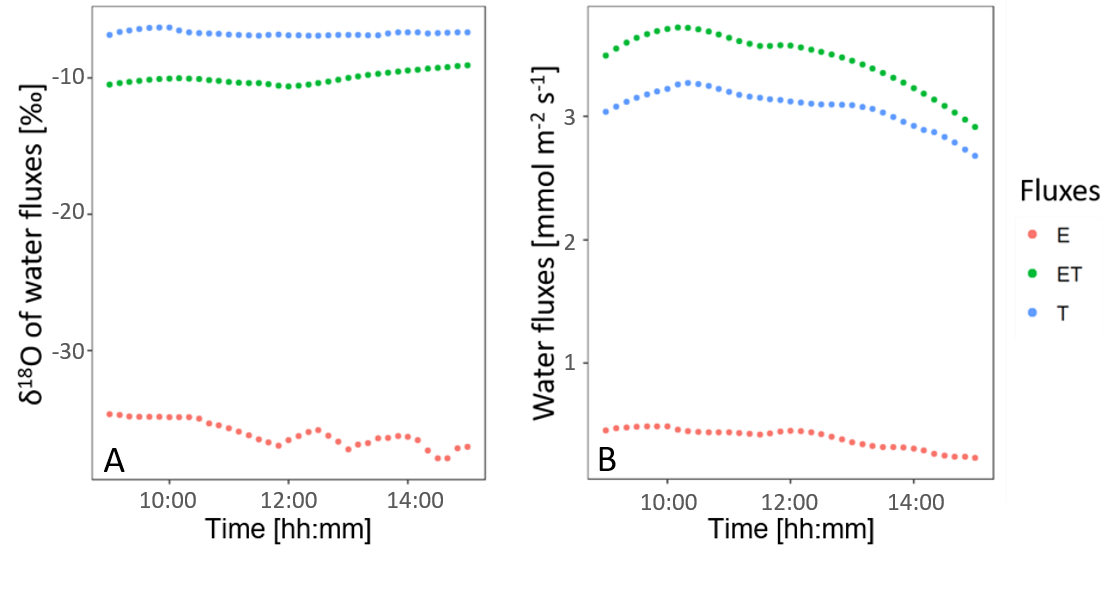


**Supplementary Figure 1**. **Example for partitioning evapotranspiration *ET***, campaign in May, 2017. (A) δ^18^O of water fluxes *E*, *T* and *ET*, in ‰. ∂*_E_*, ∂*_T_*: Mean output values of model. *∂_ET_*: Mean measured value. (B) Water fluxes, in mmol m^-2^ s^-1^. *ET*: Mean measured value. *E* and *T* according to partitioning approach.

**Appendix C: Calibrating eddy-covariance based flux data with chamber based flux data**

Supplementary Table 1. Coefficient of correlation (*r*, after Spearman) between chamber based flux measurements and eddy covariance based flux measurements, per campaign. Fluxes: Evapotranspiration (*ET*) and net ecosystem exchange (*NEE*).

| Campaign | ET | NEE |
| --- | --- | --- |
| 2017-04-21 | 0.88 | 0.88 |
| 2017-05-09 | 0.89 | 0.80 |
| 2017-05-29 | 0.88 | 0.81 |
| 2017-06-21 | 0.95 | 0.86 |
| 2017-07-04 | 0.89 | 0.82 |
| 2017-07-14 | 0.87 | 0.71 |
| 2017-07-17 | 0.84 | 0.57 |
| 2017-07-29 | 0.86 | 0.77 |
| 2017-08-16 | 0.90 | 0.84 |
| 2017-09-15 | 0.80 | 0.78 |


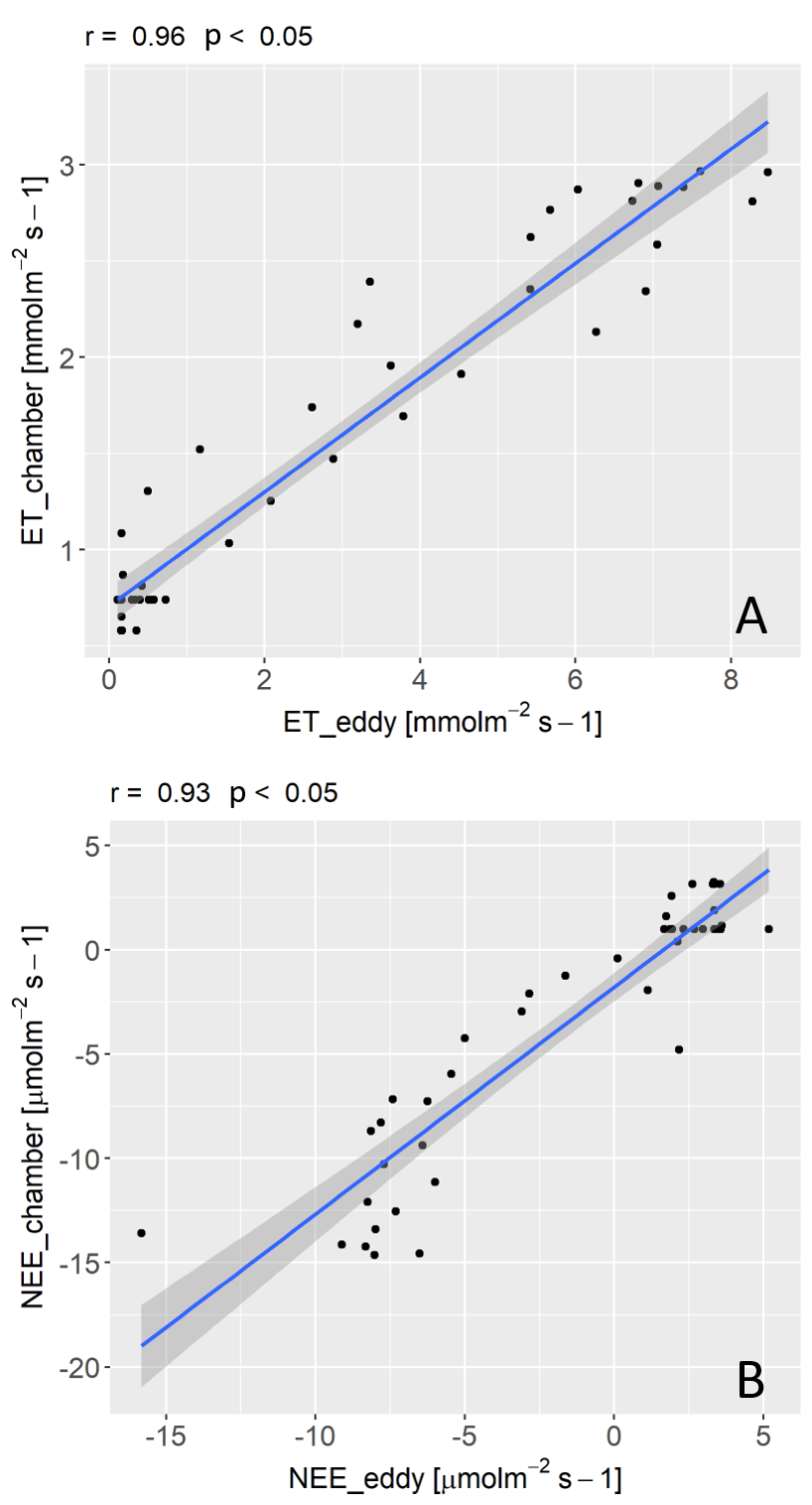


Supplementary Figure 2. Correlation between chamber based flux measurements and eddy covariance based flux measurements, example of campaign 2017-08-16. (A) Evapotranspiration *ET*.

(B) Net ecosystem exchange (*NEE*).


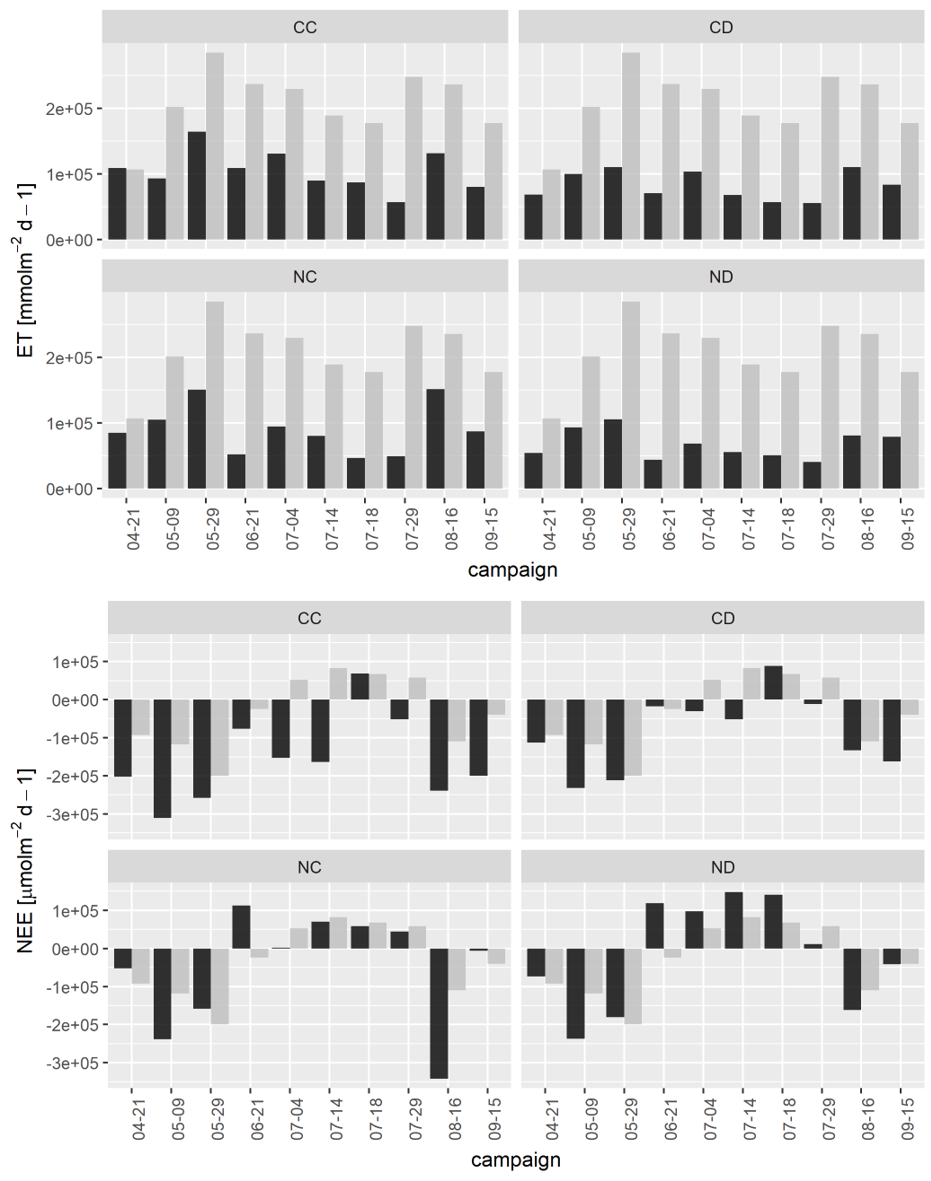


Supplementary Figure 3. Top: Daily sums of evapotranspiration *ET*. Bottom: Daily sums of net ecosystem exchange *NEE*, chamber based (black) and eddy-covariance based (grey). Per campaign and treatment. Treatments: *CC* (control, *CD* (precipitation reduction), *NC* (nitrogen addition), *ND* (precipitation reduction and nitrogen addition).


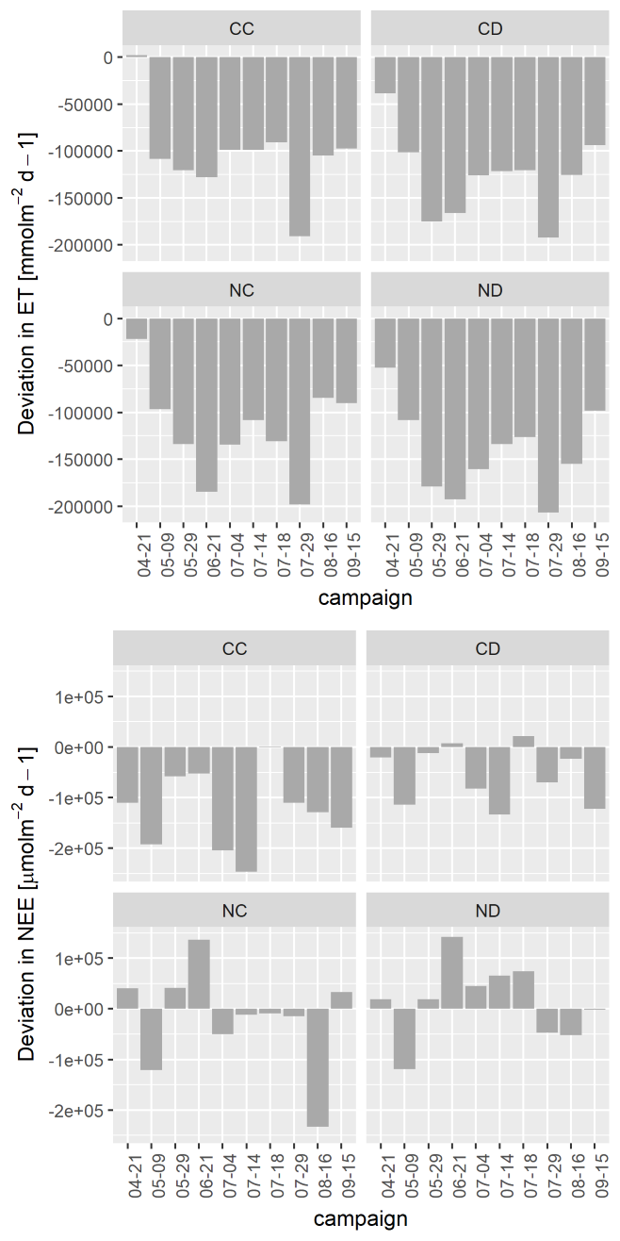


Supplementary Figure 4. Average offset for calibrating eddy-covariance based data with chamber based data, for each campaign and treatment. Top: Evapotranspiration *ET*. Bottom: Net ecosystem exchange *NEE*. After adding offset, values of *ET* an *NEE* were checked for plausibility. In very rare cases *ET* became negative, these values were removed and interpolated. Treatments: *CC* (control), *CD* (precipitation reduction), *NC* (nitrogen addition), *ND* (nitrogen addition and precipitation reduction).

Appendix D: Species list: forb species.

Supplementary Table 2. List of forb species present. This list indicates all the forb species found on the plots over the growing season 2017. They are listed from most frequently seen to least frequent.

| **Species** |
| --- |
| *Centaurea jacea* |
| *Lotus corniculatus* |
| *Achillea millefolium* |
| *Gallium mollugo* |
| *Gallium verum* |
| *Potentilla reptans* |
| *Potentilla argentae* |
| *Silene vulgaris* |
| *Plantago lanceolata* |
| *Plantago media* |
| *Thymus pulegiodes* |
| *Stellaria graminea* |
| *Hypericum perforatum* |
| *Verbena officinalis* |
| *Leucanthemum vulgare* |
| *Dianthus deltoides* |
| *Tanacetum vulgare* |
| *Cirsium arvense* |
| *Daucus carota* |
| *Trifolium repens* |
| *Hypochaeris radicata* |
| *Rumex sp.* |
| *Lathyrus platanoides* |
| *Malva ssp.* |
| *Leontodon sp.* |
| *unknown sp.* |

Appendix E: Statistical results

Supplementary Table 3. Statistical results on treatment differences for soil evaporation *E* and ecosystem respiration *R_eco_*, per measurement campaign and treatment. Treatments: *CC* (control, *CD* (precipitation reduction), *NC* (nitrogen addition), *ND* (precipitation reduction and nitrogen addition). Treatments which do not share letters are significantly different (each campaign separately, p < 0.05).

| Treatment | Date | *E* | *R_eco_* |
| --- | --- | --- | --- |
| *CC* | 20-4-2017 | a | A |
| *CD* | 20-4-2017 | b | B |
| *NC* | 20-4-2017 | c | A |
| *ND* | 20-4-2017 | d | B |
| *CC* | 8-5-2017 | ac | A |
| *CD* | 8-5-2017 | b | A |
| *NC* | 8-5-2017 | a | A |
| *ND* | 8-5-2017 | c | b |
| *CC* | 28-5-2017 | a | A |
| *CD* | 28-5-2017 | a | A |
| *NC* | 28-5-2017 | a | A |
| *ND* | 28-5-2017 | b | A |
| *CC* | 20-6-2017 | a | A |
| *CD* | 20-6-2017 | b | A |
| *NC* | 20-6-2017 | a | A |
| *ND* | 20-6-2017 | ab | a |
| *CC* | 3-7-2017 | a | a |
| *CD* | 3-7-2017 | b | ab |
| *NC* | 3-7-2017 | c | ab |
| *ND* | 3-7-2017 | d | b |
| *CC* | 13-7-2017 | a | a |
| *CD* | 13-7-2017 | b | ab |
| *NC* | 13-7-2017 | c | b |
| *ND* | 13-7-2017 | a | ab |
| *CC* | 16-7-2017 | a | a |
| *CD* | 16-7-2017 | b | a |
| *NC* | 16-7-2017 | c | a |
| *ND* | 16-7-2017 | d | a |
| *CC* | 28-7-2017 | a | a |
| *CD* | 28-7-2017 | a | a |
| *NC* | 28-7-2017 | b | a |
| *ND* | 28-7-2017 | a | a |
| *CC* | 15-8-2017 | a | ac |
| *CD* | 15-8-2017 | a | a |
| *NC* | 15-8-2017 | a | b |
| *ND* | 15-8-2017 | b | bc |
| *CC* | 14-9-2017 | a | a |
| *CD* | 14-9-2017 | b | ab |
| *NC* | 14-9-2017 | c | b |
| *ND* | 14-9-2017 | d | ab |
